# Influence of elevated temperature exposure on the residual compressive strength and radiation shielding efficiency of ordinary concrete incorporating granodiorite and ceramic powders

**DOI:** 10.1038/s41598-024-85043-2

**Published:** 2025-01-28

**Authors:** Alaa A. Mahmoud, Alaa A. El-Sayed, Ayman M. Aboraya, Islam N. Fathy, Mohamed A. Abouelnour, Maged E. Elfakharany, Mohy S. Fattouh, Abdelmoniem E. Alahmer, Islam M. Nabil

**Affiliations:** 1 Civil Engineering Department, Faculty of Engineering, Fayoum University, Fayoum, Egypt; 2 Construction and Building Engineering Department, October High Institute for Engineering & Technology, Giza, Egypt; 3 Construction and Building Engineering Department, Higher Institute of Engineering, Culture& Science City Giza, Giza, Egypt; 4 Raw Materials Department, Housing and Building National Research Center (HBRC), Giza, Egypt; 5 Civil Engineering Department, Faculty of Engineering, Sinai University, Arish, Egypt; 6 Chemistry Department, Faculty of Science, Ain Shams University, Abbassia, Cairo, 11566 Egypt; 7 Physics Department, Faculty of Science, Fayoum University, Fayoum, Egypt

**Keywords:** Construction waste, Radiation shielding, Linear attenuation coefficient, Waste granodiorite powder, Waste ceramic powder, Applied physics, Condensed-matter physics, Nuclear physics, Techniques and instrumentation, Materials science, Physics

## Abstract

This research investigates the potential of utilizing types of construction waste as partial cement replacements within concrete formulations. Notably, granodiorite and ceramic powders were introduced at varying substitution ratios. The impact of these waste materials on the compressive strength and radiation shielding effectiveness of traditional concrete was evaluated under both ambient and elevated temperature conditions. Additionally, several microstructural tests like X-ray diffraction (XRD), Thermogravimetric analysis (TGA), and Energy dispersive X-ray (EDX) were conducted to assess the influence of using the optimal replacement ratios of the investigated waste powders on the studied properties of concrete. Results revealed a substantial improvement in the investigated properties of the concrete. Remarkably, a 7% substitution with waste granodiorite powder (WGDP) yielded the optimal mix for compressive strength, exhibiting increases of 24.7%, 26.1%, 22%, and 28% at room temperature, 400 °C, 600 °C, and 800 °C, respectively. Likewise, a 7% replacement with waste ceramic powder (WCP) exhibited quantifiable improvements in compressive strength, with approximately 23.1%, 23.5%, 25.6%, and 32.6% at room temperature, 400 °C, 600 °C, and 800 °C, respectively. For microstructure analysis, XRD analysis confirmed enhanced pozzolanic activity with reduced portlandite and increased calcium silicate hydrate (CSH) formation for the optimal WGDP and WCP mixes compared to the control mix. TGA analysis revealed higher CSH decomposition in modified mixes, indicating greater pozzolanic reaction. Furthermore, density and EDX analyses showed denser microstructures in waste powders-incorporated mixes due to finer particle packing and secondary hydration effect. The radiation shielding investigation show that the optimum WCP mix (C7) enhances the attenuation capability of concrete. The optimum WGP mix (GD7) also contributes positively to attenuation, though to a lesser extent than C7. Ordinary concrete (CO) exhibits the lowest $\it \:\text{C}\text{M}$_LAC_, indicating its baseline performance in linear attenuation. *Thus, the studied CM-concrete samples provide the best protection against fast neutrons which* pave the way for the utilization of industrial waste, especially ceramic and granodiorite waste, in enhancing the properties of concrete towards radiation shielding against gamma rays and neutrons.

## Introduction

High temperature exposure has a profound influence on concrete’s performance, potentially leading to structural failure if these temperatures exceed certain thresholds or are sustained for long periods^1^. Concrete exposed to elevated temperatures experiences a range of physical and chemical changes, such as the evaporation of both free and chemically bound water, the breakdown of hydration products, the expansion of its aggregates, and an increase in porosity^2^. These changes directly cause a reduction in concrete’s mechanical properties, including compressive and flexural strength. Also, high temperatures can cause spalling due to high water vapor pressure and thermal stresses within the concrete matrix^3^. Specific temperature thresholds dictate the changes observed in concrete performance. For example, initial cracking usually starts around 600 °C, with cracks becoming more extensive and larger as temperatures approach 800 °C. Exposure to 800 °C often results in a significant decrease in compressive strength, potentially reducing it to around 50% of its original value, along with a mass loss exceeding 10% of the original concrete mass^4^. At temperatures between 1000 and 1200 °C, concrete typically loses its structural integrity, leading to significant mass loss and a substantial reduction in mechanical strength^5^.

Beyond the weakening of mechanical and physical properties, high temperatures can also impair concrete’s capacity to shield against various types of radiation. This effect intensifies as the duration of exposure to high temperatures increases^6^. Dense and well-compacted concrete acts as a powerful shield against various radiation types, including gamma rays and neutrons. However, this shielding weakens under high temperatures^7^. Extreme heat exposure can lead to cracks, increased porosity, and mass loss in concrete. The breakdown of calcium silicate hydrate (C-S-H) gel results in increased porosity and cracks, thereby reducing the concrete’s density and its effectiveness in shielding radiation^8^. Additionally, high temperatures can cause water evaporation within the concrete matrix, leading to drying shrinkage, microcracks, and a reduction in hydrogen content, which is crucial for neutron radiation attenuation^9^. The decomposition of chemical compounds like calcium hydroxide at elevated temperatures further diminishes the concrete’s capacity to scatter radiation. To mitigate the negative effects of high temperatures on the mechanical properties of concrete and its shielding capacity against radiation permeability, researchers have turned to using various materials as additives to enhance the resilience of concrete against these impacts. For example, nano-additives can be used as supplementary cementitious materials (SCMs) to improve the microstructure of concrete by activating hydration reactions or acting as fillers for the concrete’s interstitial spaces^10–12^. This can help mitigate microstructural damage and preserve the material’s strength when exposed to high temperatures Additionally, fibers or carbon tubes can be added in appropriate proportions for the same purpose^13,14^. It is also preferable for concrete exposed to high temperatures to use coarse aggregates like dolomite or basalt due to their lower thermal expansion coefficients compared to other types such as gravel and silica aggregates^15^.

Many studies have investigated suitability of waste powders in concrete. Mahmoud et al. (2024)^16^confirmed that the incorporation of marble dust and granite dust as partial cement replacements in individual mixtures improved the physical, structural, and radiological properties of ordinary concrete. Prakash et al. (2023)^17^concluded that up to 10% cement replacement with waste marble powder yielded consistent workability and a 5–10% increase in compressive strength. Notably, a 20% improvement in chloride resistance was observed with 10% marble inclusion. Rashwan et al. (2020)^18^showed that using up to 20% waste granite sludge as a cement replacement improved water penetration resistance and sulfuric acid attack resistance compared to control concrete. Other properties like water absorption, density, strength, and chloride penetration were minimally affected. The results suggest that granite sludge can be used as sustainable concrete additives up to 20% without compromising mechanical or durability performance. Du H et al. (2014)^19^examined the use of waste glass powder as a cement replacement. Up to 30% replacement did not reduce strength and improved chloride ion and water penetration resistance. At 60% replacement, these properties were significantly enhanced, with reduced electrical resistivity and water penetration depth while maintaining strength. Arif et al. (2021)^20^ found that waste brick powder improved workability but decreased density. It also enhanced strength properties due to pozzolanic reactions and a more compact microstructure.

This study investigates the suitability of waste granodiorite powder (WGDP) and waste ceramic powder (WCP) to be used as a SCM in concrete, focusing on their cementitious properties and their effectiveness in void filling. Granodiorite is a commonly used igneous rock in construction applications like flooring and facades. Its hardness has led researchers to explore its potential as a substitute for coarse or fine aggregate in concrete^21,22^. However, limited studies delve into the effects of incorporating granodiorite in powder form within concrete itself. Meziani et al. (2019)^23^investigated the impact of replacing a portion of cement with various powders, including granodiorite, in cement mortar. Their focus was on the material’s transport properties and longevity. Notably, their findings suggest a decrease in permeability when WGDP partially replaces cement, potentially improving concrete durability. This decrease implies a hindrance to fluid passage through the concrete. Conversely, the study observed an increase in the diffusion coefficient, likely linked to changes in porosity. These contrasting effects warrant further investigation into the specific mechanisms at play and the optimal utilization of WGDP in concrete mixes. Furthermore, recent research by Fathy et al. (2024)^12^suggests that partial cement replacement with finely WGDP can enhance the mechanical properties of concrete, accelerate setting times, and improve the resulting microstructure. Another recent study by Fathy et al. (2024)^24^confirmed that WGDP accelerated hydration reactions. Furthermore, increased replacement levels were found to elevate standard consistency. On the other side, the construction industry utilizes various ceramic forms, including bricks, tiles, and sanitary ware. Shaping these products often necessitates material removal through cutting, drilling, and grinding. This process generates waste in the form of dust, powder, and offcuts. Recycling WCP as a partial substitute for cement in concrete mixtures presents a promising strategy for sustainable construction. This approach not only diverts waste from landfills, promoting resource conservation and mitigating environmental impact, but it can also improve the inherent properties of concrete itself. Various studies suggest that incorporating fine WCP powder can enhance the compressive and flexural strength of concrete, with the precise effects contingent upon the specific type of WCP and the composition of the mix design^25,26^. Furthermore, recent studies have documented improvements in the high-temperature resistance of concrete reinforced with pozzolanic ceramic powder^27,28^.

The primary objective of the current study is to determine the optimal replacement ratios of the investigated waste powders with cement for enhancing concrete’s resistance against the adverse effects of high-temperature exposure for durations of two hours at temperatures of 400 °C, 600 °C, and 800 °C. The evaluation will include assessing compressive strength under both standard and elevated temperature conditions, considering replacement ratios of 1%, 3%, 5%, 7% and 9% by weight of cement. Additionally, the study will examine the concrete’s capability to attenuate various types of radiation for the optimal concrete mixes at compressive stress resistance within the investigated elevated temperatures.

## Materials and experimental study

### Materials properties

#### Cement and construction waste powders

**Fig. 1:**
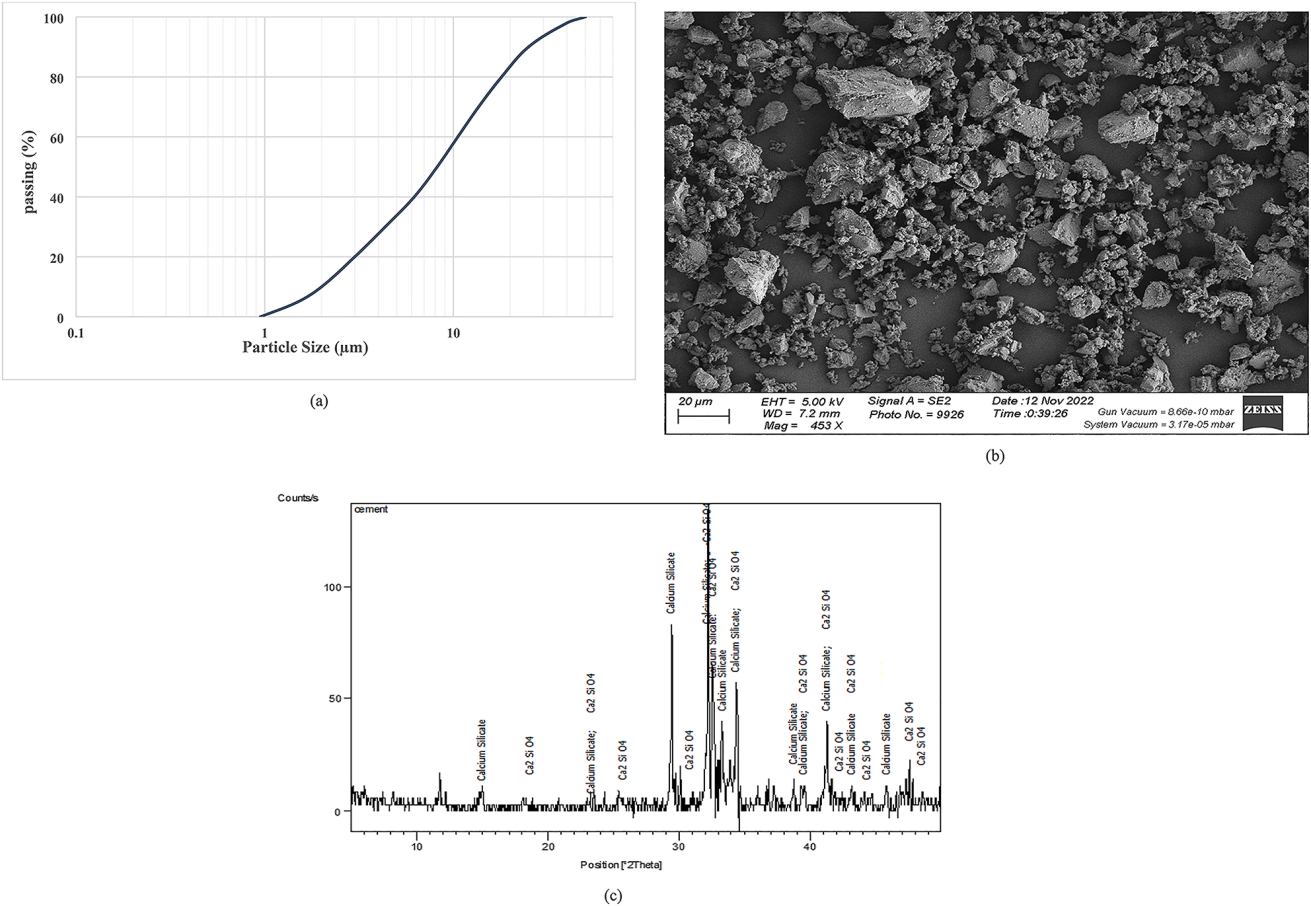
(**a**) Particle size distribution, (**b**) SEM image, and (**c**) XRD pattern of the used cement.

This investigation utilized commercially ordinary Portland cement (OPC) obtained from Wadi El-Nile company. Conforming to the ASTM C 150 standard (CEM I 42.5 N), the cement guarantees a minimum compressive strength of 42.5 MPa at 28 days. Table 1 offers a comprehensive characterization of the cement, encompassing its critical physical, chemical, and mechanical properties. The cement’s properties were further investigated, as shown in Fig. 1. The particle size distribution analysis indicates a mean particle size of approximately 8.2 μm. Additionally, the Scanning Electron Microscope (SEM) image in Fig. 1 provides valuable morphological information regarding the surface texture of the cement particles. Furthermore, the X-ray Diffraction (XRD) analysis presented in Fig. 1 provides crucial insights into the mineralogical composition of the cement. The analysis identifies calcium silicate compounds, such as calcium silicate (Ca₂SiO₄ and Ca₃SiO₅), as the primary compounds. These clinker phases are essential for the hydration process, which is responsible for the development of strength in Portland cement.

**Table 1:** Properties of the used cement and waste powders.

Physical Properties	OPC	WGDP	WCP
Color	Gray	Gray	Red
Specific gravity	3.15	2.61	2.66
Surface area(cm^2^/g)	3500	13,350	11,600
Average particle size	8.2 μm	3.5 μm	4.5 μm
Initial setting time (min)	162	-	-
Final setting time (min)	298	-	-
Mechanical			
3 days mortar compressive strength (MPa)	25	-	-
7 days mortar compressive strength (MPa)	32.5	-	-
28 days mortar compressive strength (MPa)	43.6	-	-
Chemical compositions (%)			
SiO_2_	19.82	65.3	59.40
CaO	61.72	5.28	2.89
Al_2_O_3_	3.1	16.8	15.90
Fe_2_O_3_	1.92	5.21	10.76
MgO	2.43	1.58	0.43
SO_3_	2.92	0.06	0.27
Na_2_O	0.42	1.55	1.65
K_2_O	1.17	1.27	2.83
P_2_O_5_	0.19	0.22	0.56
Cl	0.12	0.09	0.14
TiO_2_	0.38	0.38	1.83
LOI*	3.11	1.38	2.52

#### Aggregates

**Fig. 2:**
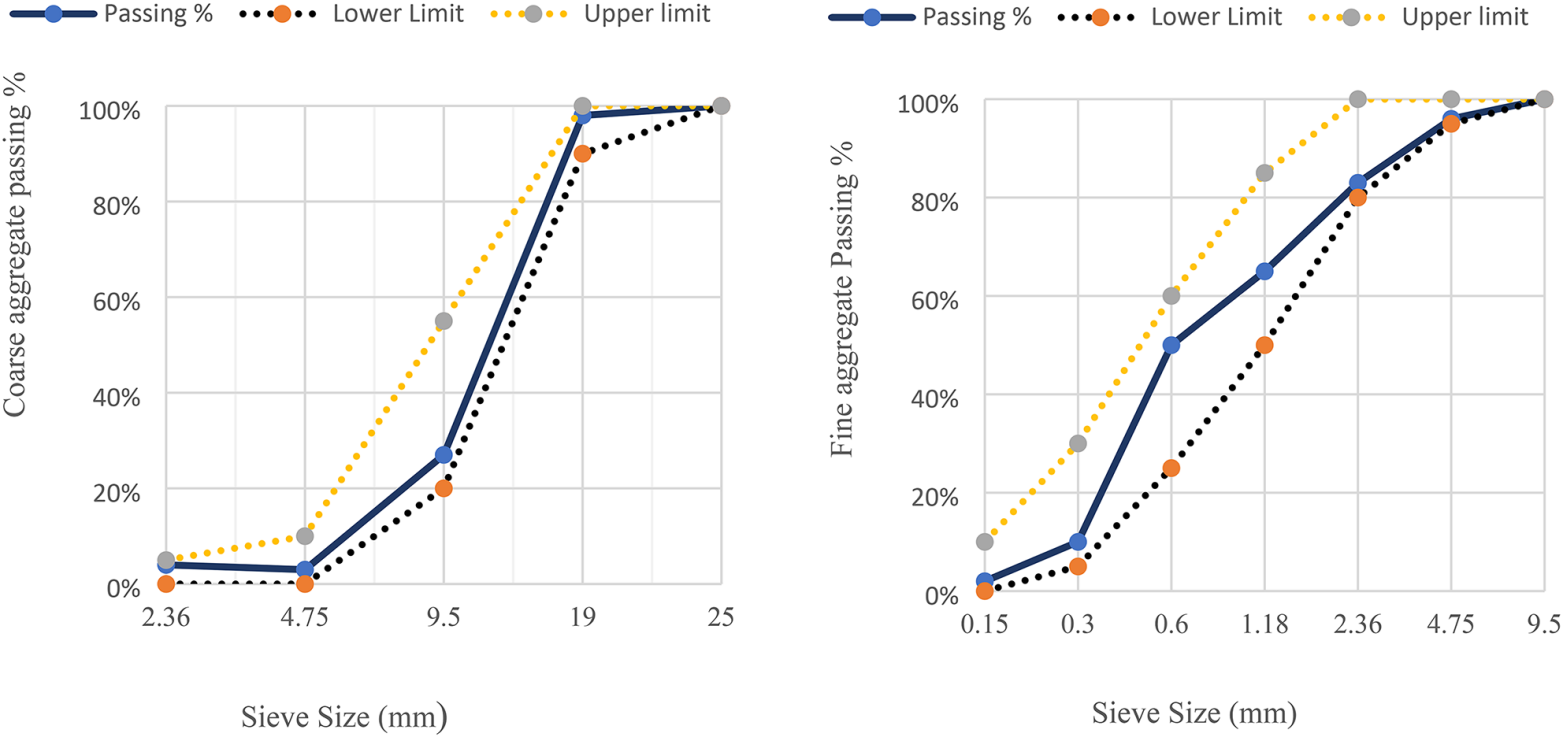
Grading of course and fine aggregates.

To ensure the coarse aggregate met the established requirements for particle size distribution as outlined in ASTM C33-03 (in Fig. 2), crushed dolomite with a maximum size of 19 mm was selected. This standard plays a critical role in guaranteeing the performance and workability of concrete by specifying appropriate limitations on the aggregate’s gradation. Selection dolomite offered a distinct advantage due to its superior thermal behavior compared to commonly used alternatives like gravel or siliceous aggregates. Notably, dolomite exhibits minimal thermal expansion and superior dimensional stability when subjected to high temperatures^15^. These properties are particularly valuable in applications where concrete structures may experience significant temperature fluctuations. Fine silica sand with a fineness modulus of 2.94 was chosen as the fine aggregate. A detailed physical property for both the coarse and fine aggregates is presented in Table 2, providing a comprehensive overview of their characteristics essential for evaluating these waste materials as sustainable alternatives in concrete.

**Table 2:** Physical properties of the used aggregates.

Aggregate type	Specific gravity	Bulk density (kg/m^3^)	Voids (%)	Absorption (%)	Clay and fine materials (%)	Crushing Value (%)	Fineness modulus
Coarse Aggregate	2.65	1690	36.2	0.45	1	19	-
Fine Aggregate	2.55	1740	31.8	0.9	1	-	2.94

#### Preparing of granodiorite and ceramic waste powders

**Fig. 3:**
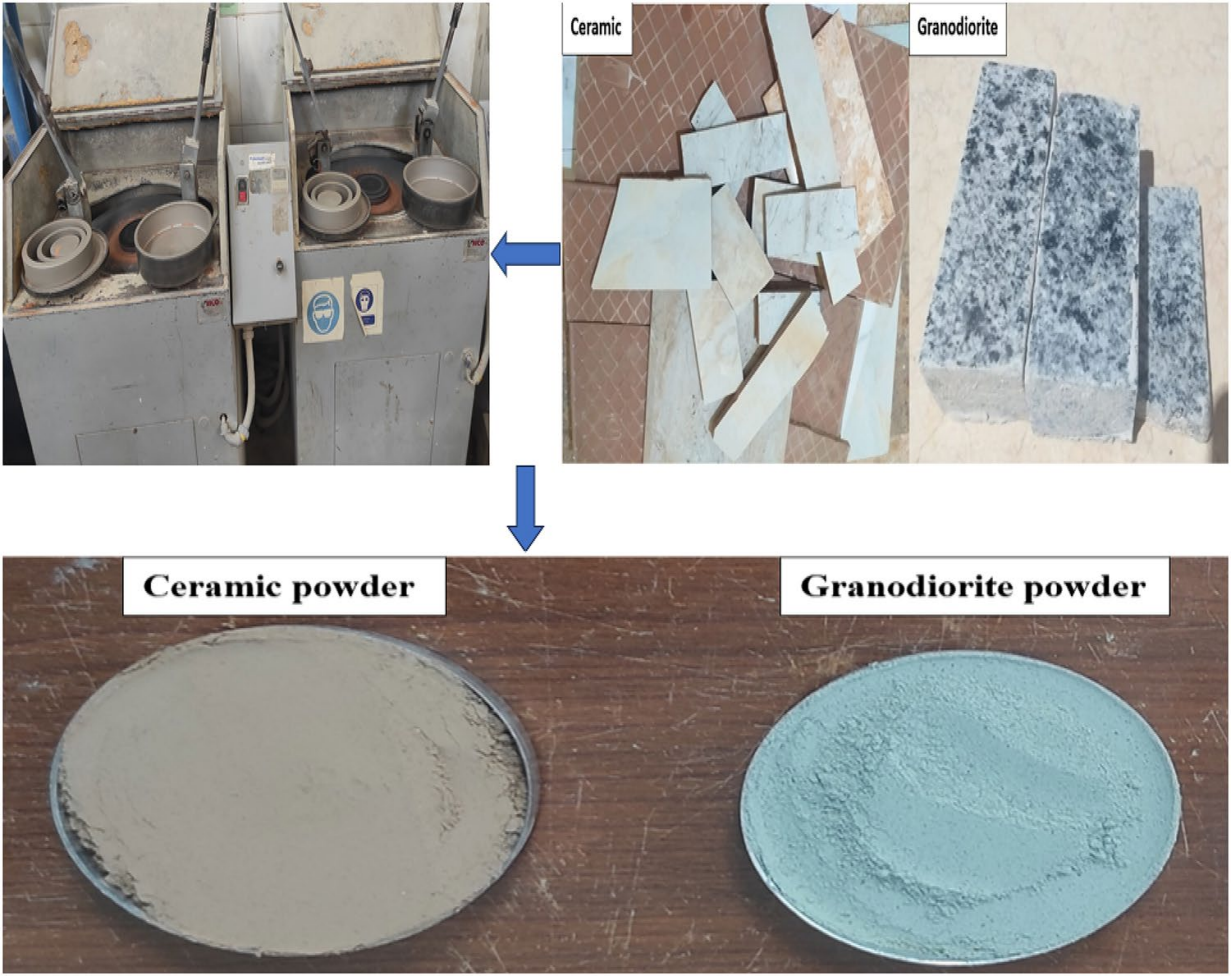
Waste construction materials powders after grinding.

**Fig. 4:**
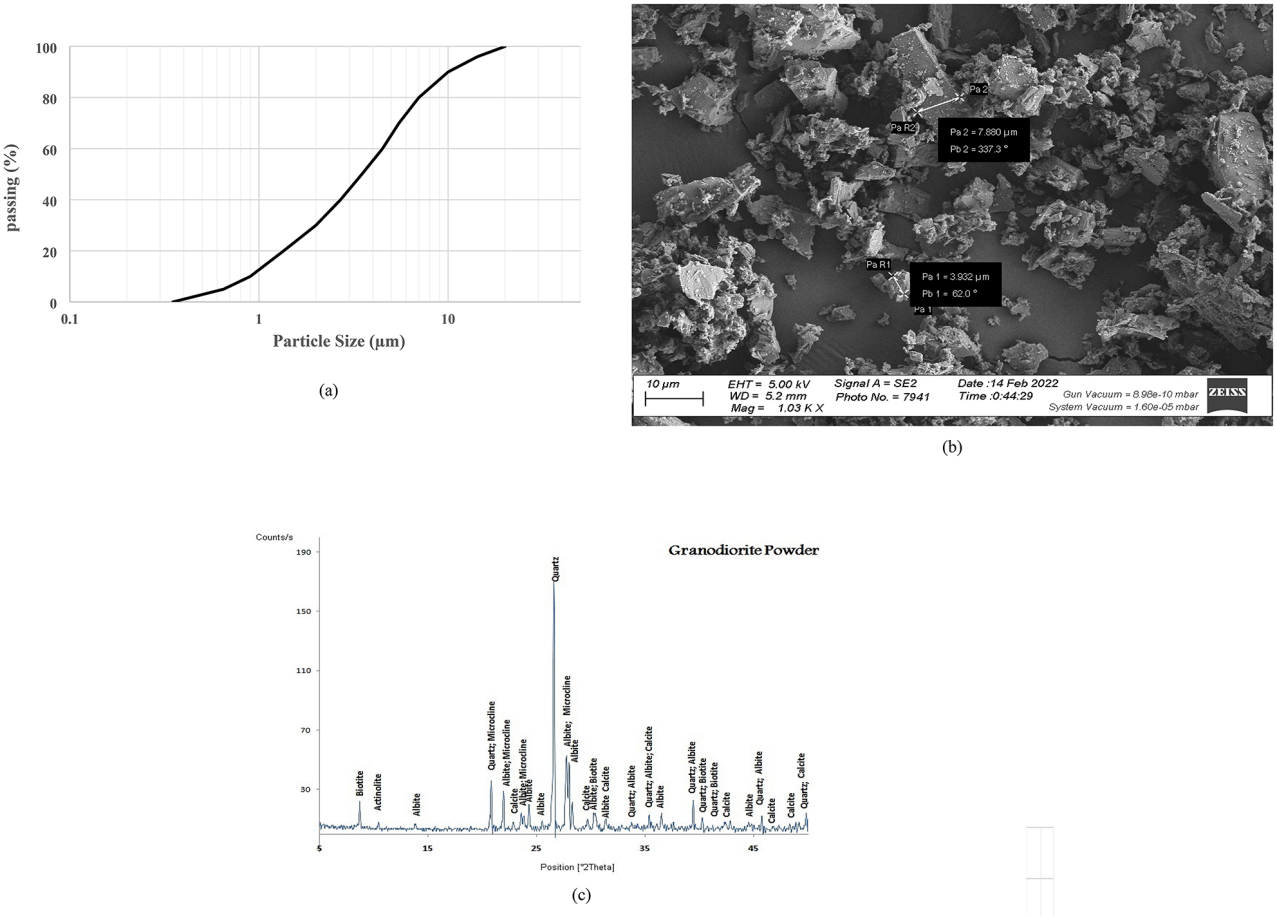
(**a**) Particle size distribution, (**b**) SEM image, and (**c**) XRD pattern of WGDP.

**Fig. 5:**
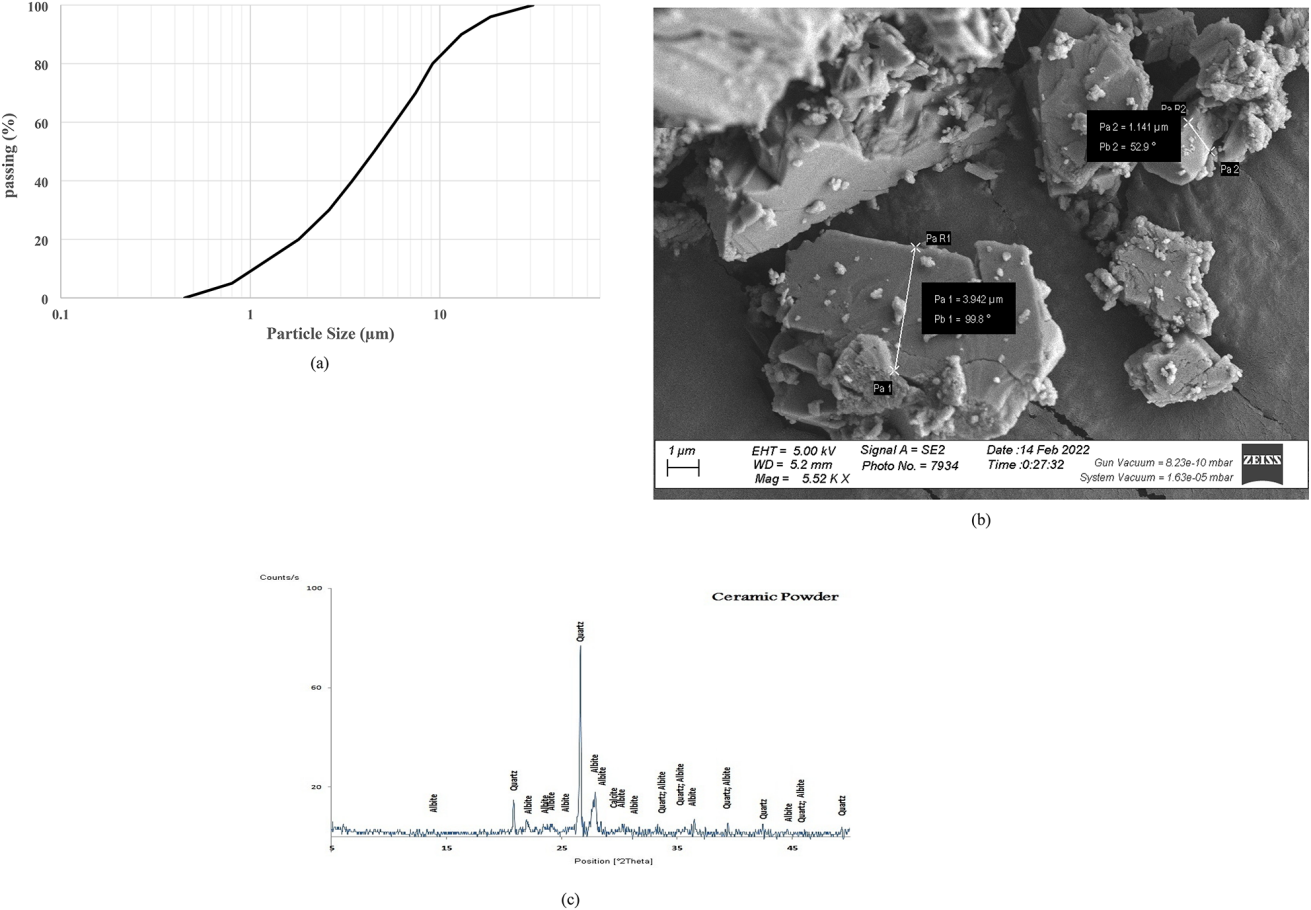
(**a**) Particle size distribution, (**b**) SEM image, and (**c**) XRD pattern of WCP.

Medium-sized waste fragments originating from the dimensional cutting of granodiorite and ceramic particles were collected for conducting the current investigation, as shown in Fig. 3. These fragments represent surplus material generated during the process of tailoring large granodiorite and ceramic pieces to desired dimensions for various applications such as facades and stairs. A vibratory pulverizer (ring mill) equipped with dual grinding rings and a grinding puck (Fig. 3) was employed to process the collected waste particles. This machine exhibits high efficiency in reducing dry mineral and rock samples to a final fineness exceeding 400 mesh within a short processing time (approximately one minute). Each sample were subjected to a standardized grinding procedure for a duration of 15 min. The resulting powders were determined to be suitable for partial cement replacement at varying incorporation ratios. WGDP and WCP were chosen due to their documented pozzolanic activity, which can contribute to improved strength development in concrete. A comprehensive characterization of the waste powders utilized in this study is presented in Figs. 4 and 5 to explain their pozzolanic behavior. Particle size distribution, SEM image, and XRD patterns are included for detailed analysis. Particle size analysis demonstrates an average diameter of approximately 3.5 μm for WGDP and 4.5 μm for WCP. These values indicate a finer particle size for both waste powders than the ordinary Portland cement (OPC) particles, which exhibit an average size of 8.2 μm, as mentioned before. This difference in particle size can significantly influence reactivity during the hydration process. The SEM images incorporated within Figs. 4 and 5 display valuable morphological information regarding the surface texture of the waste powder particles. The XRD analyses presented in Figs. 4 and 5 provide the mineralogical compositions of the waste powders. The WGDP is primarily composed of quartz (SiO₂) alongside minerals such as albite (NaAlSi₃O₈), microcline (KAlSi₃O₈), and biotite (K(Mg, Fe)₃AlSi₃O₁₀(OH)₂). The WCP consists mainly of quartz (SiO₂) and albite (NaAlSi₃O₈). Identifying the exact mineral compositions of the waste powders is critical. This knowledge directly impacts the ability to predict both pozzolanic activity (strength contribution) and the overall influence on final cementitious material properties (setting time, strength, durability).

### Design of concrete mix

This study investigates the impact of incorporating waste construction material powders, namely WGDP and WCP, on the compressive strength and radiation shielding effectiveness of conventional concrete. Substitution levels for each waste material were systematically increased: 1%, 3%, 5%, 7%, and 9% by weight of cementitious materials. The reference concrete mix design adhered to the specifications outlined in the ACI 211.1 standards. The composition of a cubic meter of the reference mix (Table 3) comprised 400 kg of Portland cement, 1165 kg of coarse aggregate, 645 kg of fine aggregate, and 180 kg of water. This yielded a fixed water-to-cement (w/c) ratio of approximately 0.45. To achieve adequate workability while maintaining the low w/c ratio (essential for strength development), a high-range water reducer (HWR) was employed. The dosage of the HWR, commercially named HYMIX G-500, was 0.60% by weight of cementitious materials, conforming to the manufacturer’s recommended range (0.60 − 1.80%). HYMIX G-500 is a polycarboxylate-based HWR (ASTM C494, Type G) with a specific gravity of 1.16 kg/L, a pH of 5.8, and a solid content of 30%. The decision to incorporate an HWR was driven by the anticipated challenges of effectively mixing the high surface area waste powders and maintaining workability at the chosen w/c ratio.

**Table 3:** Concrete mix design (kg/m^3^).

Mix	Symbol	Cement	WGDP	WCP	Aggregate (kg)		Water	HWR
		(kg)	(kg)	(kg)	Coarse	Fine	(kg)	(kg)
Control	C0	400	-	-	1165	645	180	2.4
1% WGDP	GD1	396	4	-	1165	645	180	2.4
3% WGDP	GD3	388	12	-	1165	645	180	2.4
5% WGDP	GD5	380	20	-	1165	645	180	2.4
7% WGDP	GD7	372	28	-	1165	645	180	2.4
9% WGDP	GD9	364	36	-	1165	645	180	2.4
1% WCP	C1	396	-	4	1165	645	180	2.4
3% WCP	C3	388	-	12	1165	645	180	2.4
5% WCP	C5	380	-	20	1165	645	180	2.4
7% WCP	C7	372	-	28	1165	645	180	2.4
9% WCP	C9	364	-	36	1165	645	180	2.4

### Mixing, casting and curing of concrete specimens

This investigation employed cubic steel molds with standardized dimensions of 10 cm x 10 cm x 10 cm to cast concrete specimens and evaluate the effects of waste powder incorporation on their compressive strength and radiation shielding properties. Coarse and fine aggregates were dry-mixed for a duration of three minutes. Subsequently, cement and waste powder were introduced and blended for an additional two minutes. Finally, a pre-measured quantity of water containing the superplasticizer admixture, in a dissolved state, was incorporated and mechanically mixed for another two minutes. To ensure optimal compaction and eliminate entrapped air, the fresh concrete was vibrated using an electric vibrator after being cast into the molds. The mold surfaces were then meticulously smoothed. Following demolding at the 24-hour mark, the specimens underwent a curing process in a controlled environment maintained at a temperature of 25 ± 2 °C with a relative humidity exceeding 95% for a designated testing period of 28 days.

### Testing procedures

**Fig. 6:**
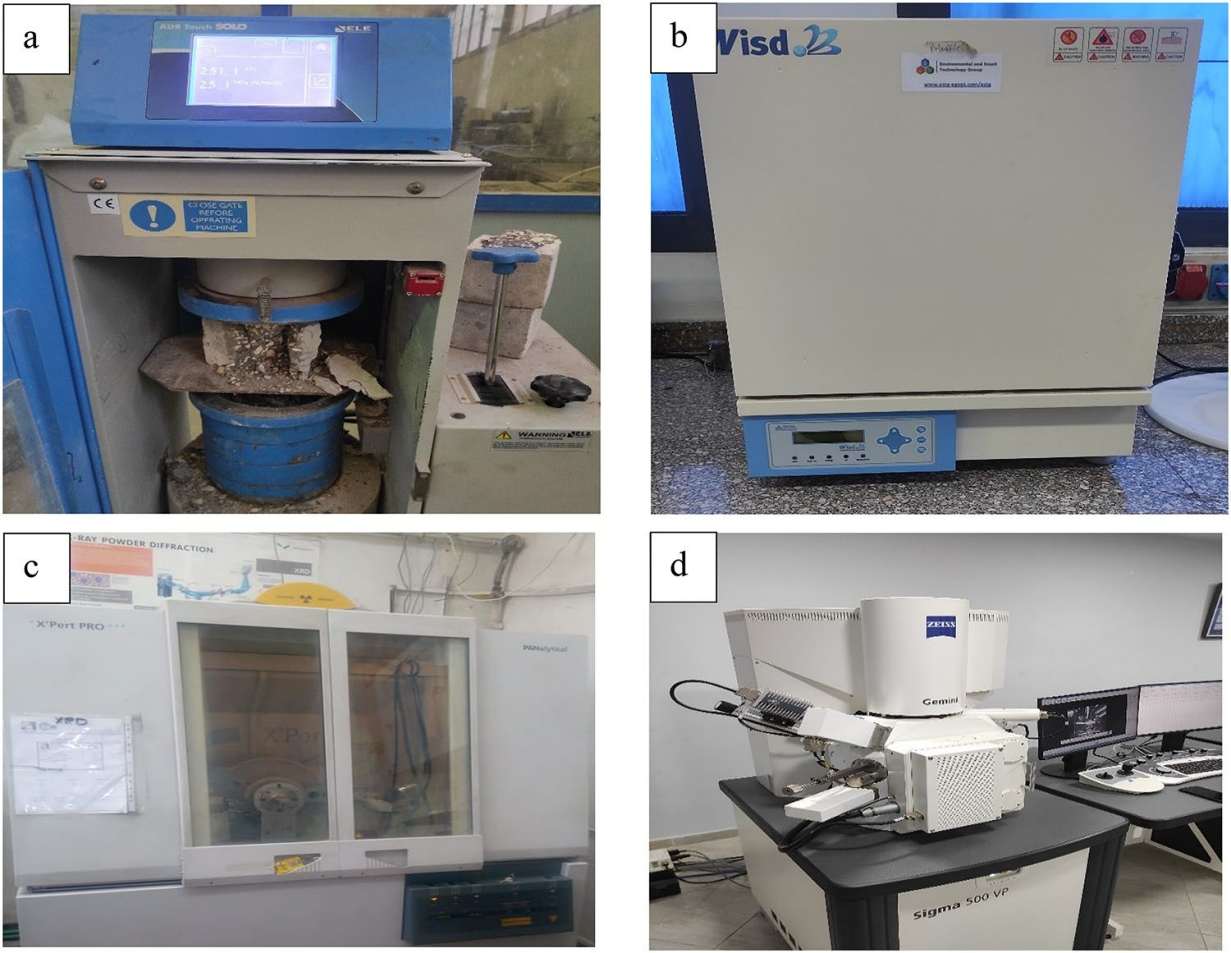
(**a**) Compressive strength testing apparatus (**b**) the electrical furnace, (**c**) XRD apparatus, and (**d**) EDX apparatus.

Compressive strength is a fundamental mechanical property of concrete to represent its overall mechanical strength. In this study, the values of compressive strength of the investigated specimens were assessed after a 28-day curing period. Compressive strength measurements were captured using a uniaxial compression testing apparatus capable of exerting a maximum load of 3000 kN (Fig. 6a). Concrete cubes, each with a side length of 10 cm, were horizontally positioned within the testing apparatus and subjected to continuous compressive force until failure.

To elucidate the impact of elevated temperatures on the residual compressive strength of concrete specimens formulated with WGDP and WCP, a muffle furnace of model (Wisd) capable of achieving exceptionally high temperatures was employed (Fig. 6b). This furnace boasts a maximum operating temperature of 1200 °C, exceeding the requirements of this investigation. The chamber dimensions of 400 × 300 × 300 mm offer ample space for the simultaneous heating of four concrete cubes per heating cycle. The furnace prioritizes operator safety during operation through its high-temperature-resistant construction and an additional insulation layer, ensuring efficient heat retention within the chamber. A comprehensive microstructural analysis of the concrete specimens was conducted using a suite of advanced techniques: XRD, EDX, and Thermogravimetric Analysis (TGA). XRD (Fig. 6c) is a non-destructive technique that identifies the crystalline phases present in a material by analyzing its unique diffraction pattern. The information obtained from XRD was used to corroborate the chemical composition of the WGDP and WCP as determined by X-ray Fluorescence (XRF) (Table 1). Additionally, XRD aided in verifying the expected amorphous state of these waste materials, a characteristic not readily detectable by XRF. On the other side, EDX (Fig. 6d) provides elemental analysis at the microscopic level, enabling the identification and quantification of elements within a localized region of the sample. This technique is particularly valuable for pinpointing the distribution of elements within the hydrated cement matrix and the waste particles themselves. In conclusion, TGA is a technique that determines the change in mass of a material as a function of temperature. Quantification of the amount of water that is chemically bound within the hydrated cement paste of concrete can be accomplished through the utilization of TGA, which enables an assessment of the hydration reaction to take place. When the amount of calcium hydroxide (CH) measured through TGA is lower, it indicates that the hydration reaction is more complete. This, in turn, generally translates to the concrete having greater strength and durability^29^. In this context, TGA can be employed to assess the thermal decomposition processes occurring within the concrete specimens, potentially revealing information regarding the presence of hydrates or other thermally sensitive components.

### Radiation shielding measurements

#### MCS5 code

**Fig. 7:**
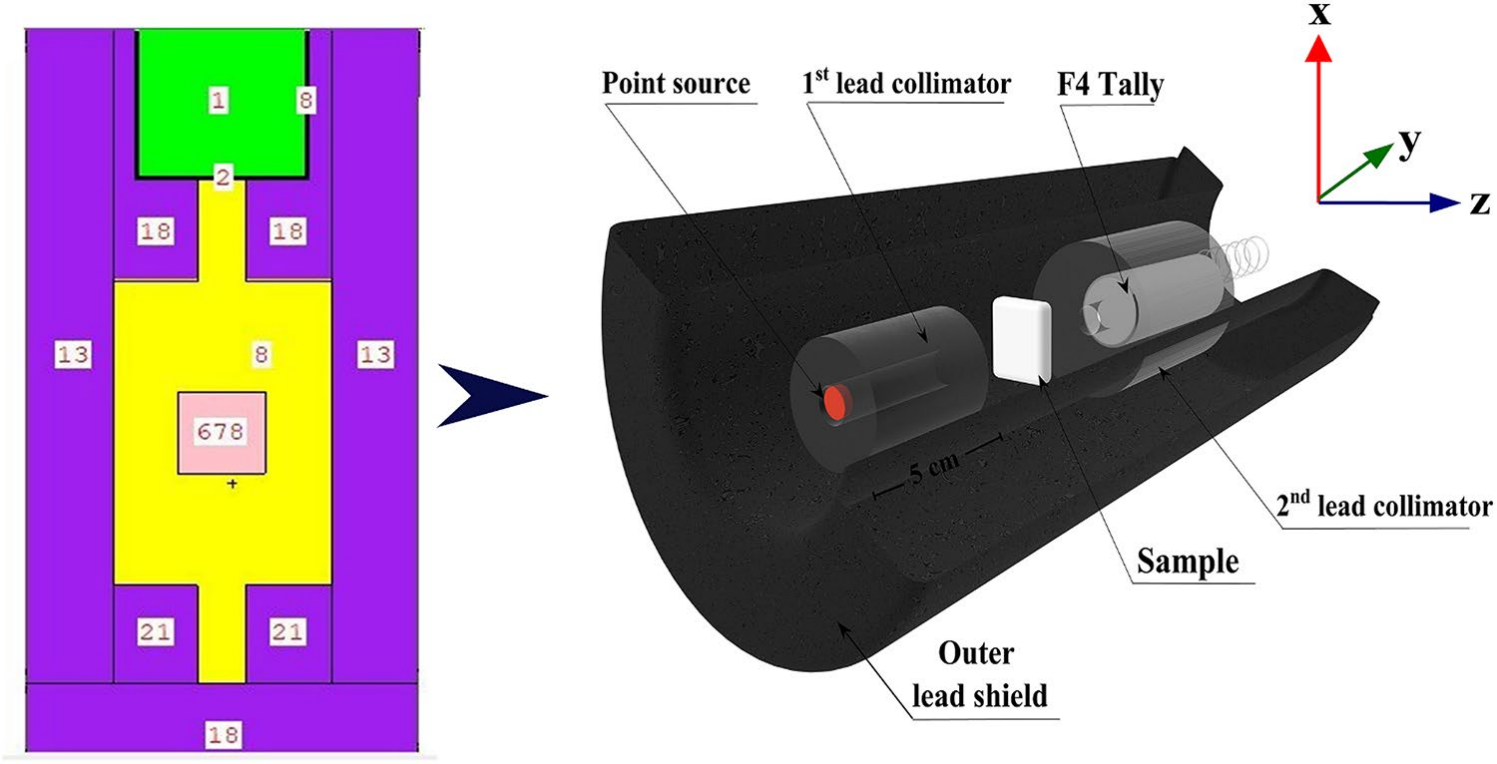
3-D view of the radiation attenuation simulation system used for the synthetic concrete samples.

The code was developed with the express purpose of imitating the behavior of natural particles through the application of the Monte Carlo method. With the help of the MCS simulation system, the theoretical intensity of γ-rays that are emitted by γ-point sources was properly predicted. Within the photon energy range of 0.015 to 15 MeVs, the simulation utilized a source of γ-emitter that possessed a relatively high level of energy. A comparison of the gamma-ray intensity before and after it has passed through the concretes that are the subject of the investigation is the goal of this endeavor. MCS codes are frequently preferred in research investigations, including those involving radiation shielding and safety, dose calculation, and detector design, among other types of investigations^30^. The preference for these codes is due to their array of advantageous characteristics, including their ability to function across a wide range of energy levels, their flexibility in accommodating different geometrical designs, and their ability to perform rapid calculations. The objective of this technology is to improve the mobility of electrons, neutrons, and γ-rays by taking into account a variety of mechanisms that participate in the interaction of photons^31–33^. In order to conduct an MCS simulation, it is imperative to include accurate details in the input file pertaining to the geometry, distance between the source and detector, dimensions of the source (S-D-E-F source card), as well as the elemental and chemical composition and densities of the concrete samples being examined (material card)^34–36^. Fig. 7 illustrates how the geometric configuration of the simulation was designed by making use of a pre-existing two-dimensional and three-dimensional setup. Each and every parameter has been tested and found to be consistent with the experimental system. The text lines that were used to create the input files for the MCS simulation were created. A radioactive source, primary and secondary γ-radiation collimators, a sample comprising a cubic shape, and a detector were the six distinct components that comprised the cell at the time of its construction. The identification of a point source of γ-rays was accomplished by identifying a single, well-defined, and monoenergetic flow of γ-rays for every input file. The γ-ray energies are up to 15 MeV^37^. The neutron source is defined as a californium spectrum that operates within the energy range of 0 to 11 MeV, allowing for efficient elimination of σ attenuation for fast neutrons. These specimens were manufactured in the form of a layer that was cylindrical in shape. In addition, the material card of the TXT coded lines was used to record the measurements of the specimens’ densities as well as the elemental composition of the samples. In order to detect secondary γ-rays, the detector was positioned inside a lead collimator that was specifically designed for the purpose. The Tally command is responsible for determining the sum of all the values that fall inside the F4P range. At the same time, the F4N algorithm calculates the average length of the γ-rays and neutrons that are emitted by simulated γ/neutron radiation sources. An outer shield composed of lead was utilized in order to encompass the created detector, collimators, source, and specimens that were being investigated that were being examined. The computations were carried out with the assistance of a core i-5. More than one N-P-S (12^7^) attempts were conducted for each file to ensure that random statistical errors remained below 1%^33^. The total runtime for the calculations involving (260) input files was approximately 10.6 min per run.

The radiation performance of the materials can be described using a variety of attenuation coefficients which are as follows^38,39^:

1 $${\text{I}}\,=\,{{\text{I}}_{\text{o}}}{\text{exp}}( - {\text{C}}{{\text{M}}_{{\text{LAC}}}}{\text{X}})$$

2 $$\:{\text{C}\text{M}}_{\text{M}\text{A}\text{C}}=\frac{\text{C}\text{M}_{\text{L}\text{A}\text{C}}}{\rho\:}$$

3 $$\:{\text{C}\text{M}}_{\text{H}\text{V}\text{L}}=\:\frac{\text{l}\text{n}2}{\text{C}\text{M}_{\text{L}\text{A}\text{C}}}\:\:\:\:$$

4 $$\:{\text{C}\text{M}}_{\text{T}\text{V}\text{L}}=\:3.32\:{\text{C}\text{M}}_{\text{H}\text{V}\text{L}}$$

5 $$\:{\text{C}\text{M}}_{\text{M}\text{P}\text{F}}=\frac{1}{\text{C}\text{M}_{\text{L}\text{A}\text{C}}}\:\:\:\:$$

I denote the magnitude of the γ-rays that have penetrated the material. The mass attenuation (CM_MAC_), the half value layer (CM_HVL_), the tenth value layer (CM_TVL_), the mean free path (CM_MPF_), and the intensity of the main γ without the material are all represented by I_o_. In contrast, X represents the thickness of the material.

Also, the neutron attenuation potential of suggested materials may be determined by calculating the fast neutron removal cross-section (CM_FC_) using the equation CM_FC_ =$\:\sum\:_{i}{W}_{i}$ x ρ, $\:{W}_{i}\:$(the partial density), ρ (the material density), and i (the mass cross-section of the component).

#### Phy-X software

The Phy-X/PSD software (PhyX) was employed to validate the results of the MCS model^40^. The discrepancies (Δ, %) were also determined by comparing the Phy results for the CM samples with the MCS results:

6 $$\:{\Delta\:}\:\left(\text{\%}\right)\:=|\frac{MCS\:-PhyX}{MCS}|\times\:100$$

## Results and discussion

### Mechanical properties

#### Compressive strength at traditional conditions

**Fig. 8:**
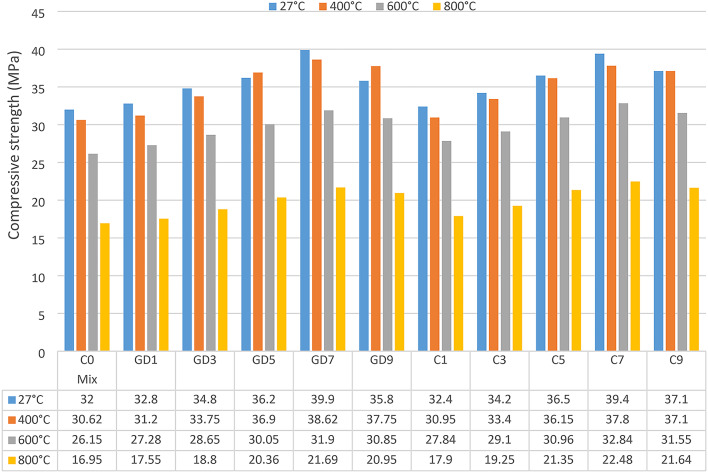
28-day compressive strength of all mixes at room and elevated temperatures.

Concrete specimens incorporating both WGDP and WCP exhibited significant improvements in compressive strength across all replacement ratios, as demonstrated in Fig. 8. Strength exhibited a steady upward trend with increasing waste powder content, reaching peak enhancements of nearly 23.1% and 24.7% at a 7% replacement ratio for WGDP and WCP, respectively. This phenomenon can be attributed to the multifaceted mechanisms associated with the physical and chemical properties of these waste materials. The fine waste additive particles effectively fill microstructural voids within the concrete matrix, resulting in a denser and more robust overall structure. This densification reduces porosity, a key factor influencing concrete strength. Lower porosity minimizes the formation of stress concentration points within the concrete, leading to a higher capacity to withstand compressive loads. Furthermore, the chemical composition of the waste additives plays a vital role. Rich in amorphous silicates, aluminates, and calcium oxides (75–82% according to XRF analysis), both WGDP and WCP can act as pozzolanic materials. The high silica content in WGDP and WCP can react with calcium hydroxide (CH) released during cement hydration, forming additional C-S-H gels. This pozzolanic reaction consumes excess CH and promotes further hydration. While this is a simplified explanation, the underlying mechanism is more complex, involving the polymerization of alkali-activated aluminosilicate gels, similar to geopolymer concrete^41,42^. However, as hydration progressed, the free water in the paste transformed into adsorbed and bound water. The increasing formation of CSH gel limited the availability of polar water molecules, hindering their ability to polarize the cement particles^43^. The formation of more CSH through the pozzolanic reaction reinforces the concrete matrix, enhancing its ability to resist compressive forces. While the lower specific gravity of WGDP and WCP can initially reduce concrete density, this effect is mitigated by the filler effect. Their finer particle size and higher surface area, compared to cement particles, allow them to fill voids between larger cement particles, leading to a denser matrix. Additionally, the larger surface area of WGDP and WCP can enhance their reactivity with water, promoting stronger bonds with hydration products during the hydration process and improving concrete strength, including compressive strength^44,45^. Notably, the incorporation of WGDP and WCP not only enhances compressive strength but also avoids any detrimental effects on the material at the optimal replacement ratio of 7%. This aligns with observations reported in previous research^46,47^, underlining the consistent and effective role of waste powders in improving concrete strength. However, exceeding this optimal ratio could potentially lead to negative effects, such as introducing excessive voids or hindering proper hydration due to a higher water demand from the pozzolanic reaction.

#### Compressive strength at elevated temperatures

**Fig. 9:**
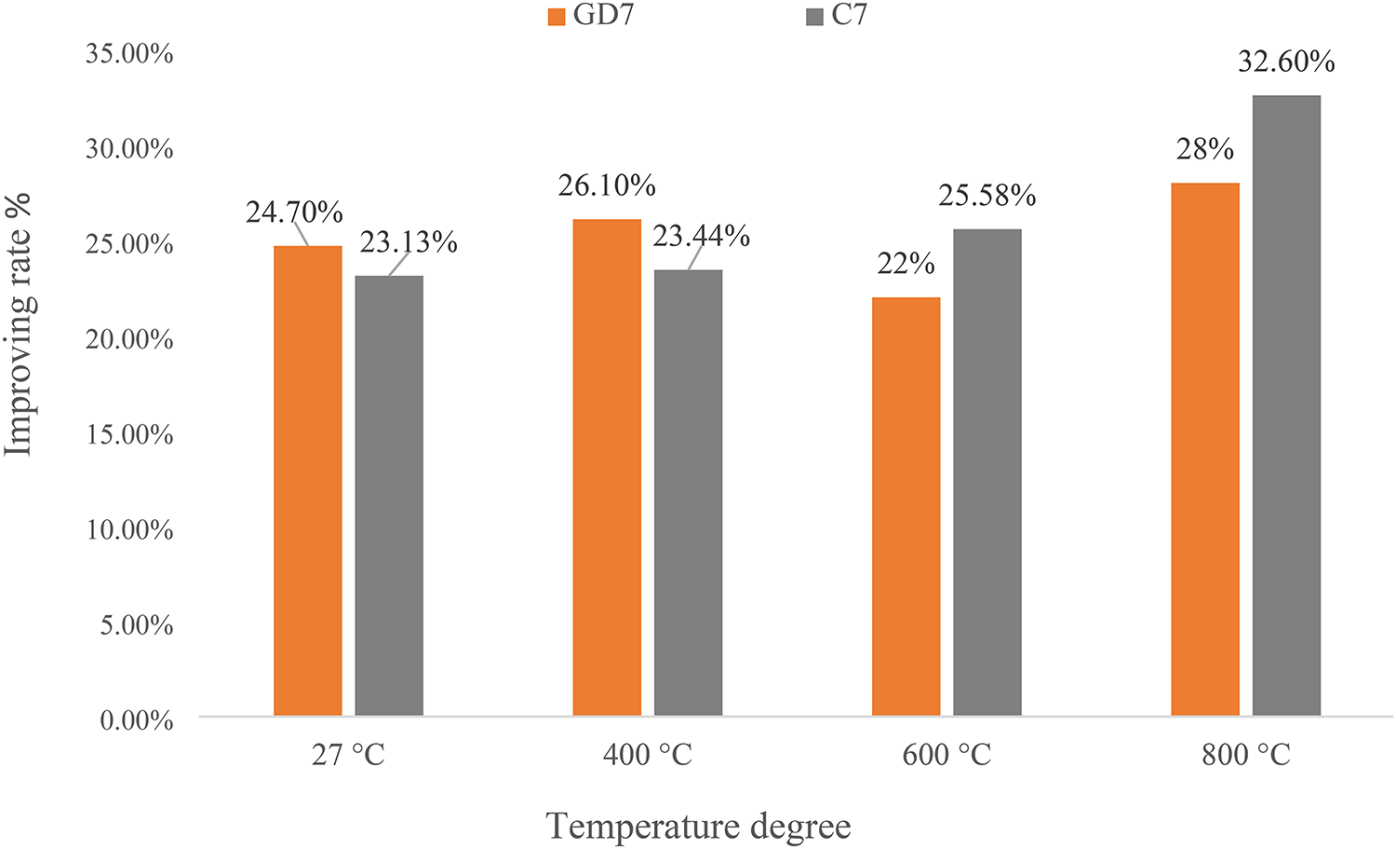
Residual compressive strength improving rates for optimum mixes at elevated temperatures.

To comprehensively evaluate the combined influence of elevated temperatures on both the residual compressive strength and shielding effectiveness, samples from the control mix and all designed mixes were subjected to a progressive thermal exposure regimen, encompassing temperatures of 400 °C, 600 °C, and 800 °C, maintained for a two-hour exposure duration. Fig. 8 illustrates the compressive strength of all designed mixes following thermal exposure. Elevated temperatures exert a profound impact on the intricate microstructure of cementitious materials, inducing detrimental transformations beyond critical thresholds that expose their structural safety. A primary consequence is the dehydration of hydration byproducts such as CSH and CH^48^. This liberates water, disrupting the network of interlocking crystals that underpins strength and durability. The resultant microcracks function as stress concentrators, further weakening the material. Exacerbating this deterioration is the decomposition of cement minerals, leading to the formation of additional pores and heightened porosity. This amplified porosity renders the cement paste more susceptible to crumbling. Additionally, thermal exposure induces the conversion of CH into calcium oxide (CaO), further contributing to microcrack formation. Ordinary concrete experiences significant degradation in mechanical properties when exposed to extreme temperatures exceeding 600 °C^49^. This triggers chemical reactions that generate pressurizing gases within the binder network, causing extensive cracking and a substantial reduction in compressive strength (potentially exceeding 50%), potentially leading to catastrophic consequences like structural failure. This investigation delves into the efficacy of incorporating two waste construction powders at optimal replacement ratios to enhance concrete’s residual compressive strength after high-temperature exposure. Fig. 9 demonstrably depicts a clear correlation between waste powder replacement ratios and improvements in residual compressive strength. Mixes GD7 and C7 exhibited a remarkable increase in residual compressive strength relative to the control mix following exposure to high temperatures. When compared to the values of the control mix, the residual compressive strength values of the GD7 and C7 mixes improved by 28.0% and 32.6%, respectively, after being subjected to a temperature of 800 °C. Among the most important mechanisms is the pozzolanic reaction that takes place between SCMs and CH. C-S-H and calcium alumino-silicate hydrates are two examples of the extra cementitious hydrates that are formed as a result of this reaction, which takes place within the cement matrix. Additionally, the newly produced C-S-H gel is able to effectively fill gaps and spaces, which results in a microstructure that is denser. The concrete’s resistance to heat-induced damage is improved as a result of this increased density, and its permeability is additionally decreased. When subjected to thermal stress, the added hydration products operate as a mitigating element, thereby preventing the formation of cracks. Improved spalling resistance and superior residual compressive strength values are achieved by concretes that have been treated to elevated temperatures as a result of the combined effects of SCM incorporation, which include pore refinement and greater hydration^50^.

### Microstructural properties

#### XRD results

**Fig. 10:**
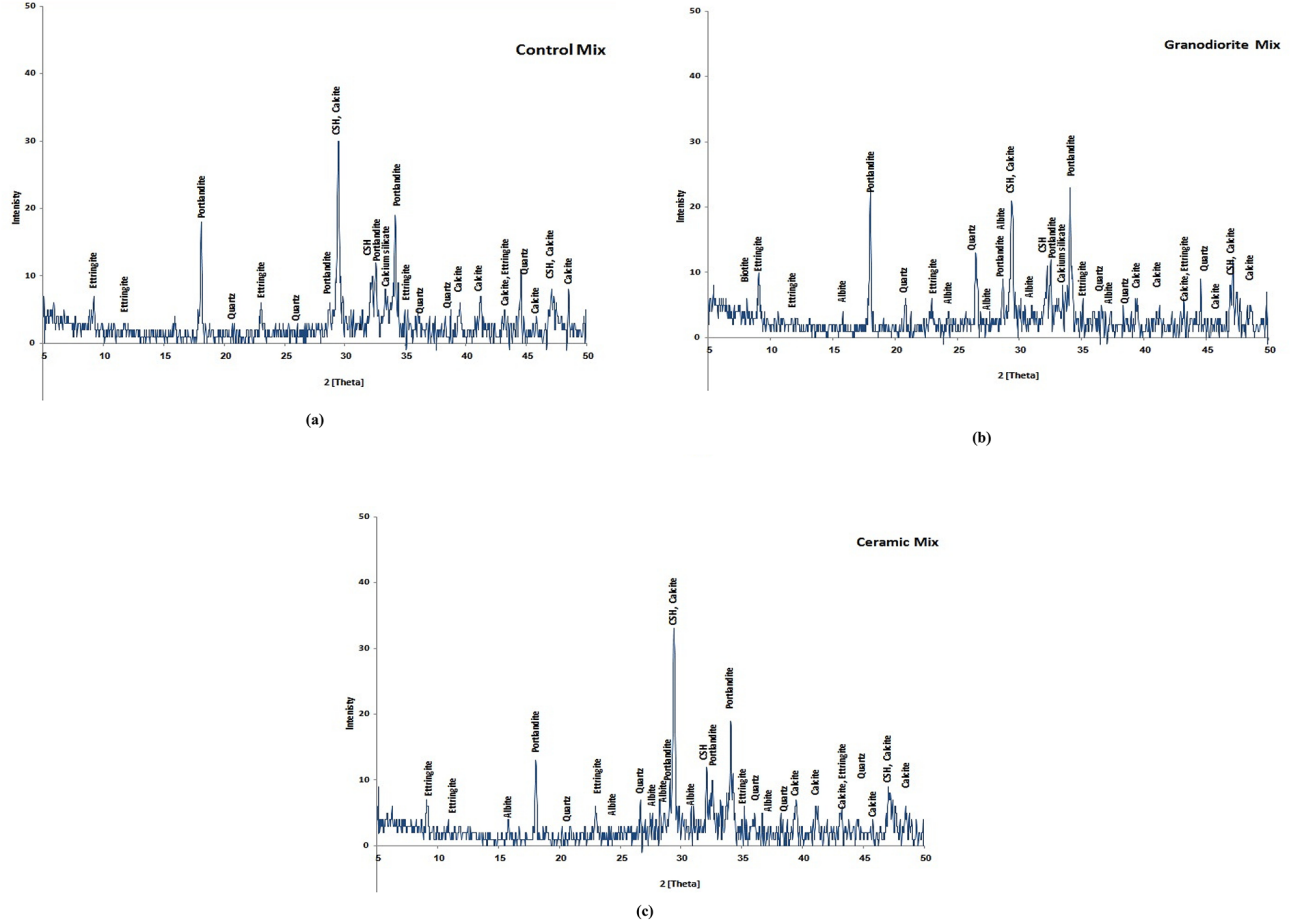
XRD patterns for (**a**) the control, (**b**) 7%WGDP, and (**c**) 7% WCP cement pastes.

XRD analysis was utilized to explain the influence of waste powders on the composition, hydration processes, and microstructure of cement pastes. Three cement pastes were investigated: a control paste (CO) and pastes containing optimal replacement ratios of granodiorite powder (GD7) and ceramic powder (C7), as determined by strength tests. As expected, XRD successfully identified calcium silicate hydrate (CSH) and portlandite (CH) as the principal hydration products in all pastes, confirming their roles in setting and strength. Additionally, quartz, a common mineral component in both WGDP and WCP, was detected through XRD analysis. The intensity of each peak in the XRD pattern reflects the mount of formed compounds as the increase in intensity refers to the high in the amount of compound. As displayed in Fig. 10, the main and secondary peaks of CSH were identified at 2 Theta of 29.36° and 32.3°, respectively. The main and secondary peaks of portlandite were identified at 2 Theta of 34.1° and 18°, respectively. The peak of quartz was identified at 2 Theta of 26.6°.

The findings from the XRD analysis, presented in Fig. 10, provide evidence that aligns perfectly with the previously observed improvements in mechanical properties of the cementitious pastes containing pozzolanic powders (GD7 and C7). XRD analysis provides valuable insights into the reaction mechanisms occurring within the pozzolanic pastes. A noteworthy observation is the decrease in the intensity of portlandite (CH) peaks for both GD7 and C7 compared to the control. The observed decrease in portlandite (CH) peak intensity suggests the pozzolanic powders actively participated in the hydration process. Pozzolanic WGDP and WCP, typically rich in silicate (SiO₂) primarily in the form of quartz, consume CH during hydration. This pozzolanic activity aligns with established findings reported by Abouelnour et al. (2024)^51^, where pozzolanic reactions promote the conversion of CH, a readily available byproduct of cement hydration, into additional calcium silicate hydrate (CSH) gel. This consumption of CH by the pozzolanic powders effectively transforms a less desirable hydration product CH into a more favorable and mechanically beneficial phase CSH. This preferential formation of CSH is directly reflected in the increased intensity of CSH peaks observed in the XRD analysis.

Interestingly, the intensity of the quartz peak exhibited significant variation between the pozzolanic pastes. Granodiorite paste (GD7) displayed a considerably higher presence of unreacted quartz compared to the ceramic paste (C7). This observation necessitates a closer examination of two potential explanations. One explanation focuses on the possibility of a more efficient pozzolanic reaction mechanism within the ceramic paste. Pozzolanic materials, like WCP in this case, react with the CH liberated during cement hydration to form additional CSH. This increased consumption of silica, primarily present in the form of quartz (SiO₂), would translate to a lower abundance of unreacted quartz observed in the XRD pattern of the C7 paste compared to GD7. Alternatively, the observed disparity in quartz peak intensity could be attributed to inherent differences in the initial chemical composition of WGDP and WCP. The ceramic powder might inherently possess a lower initial concentration of silicates compared to GD7. This lower starting point for available silica within the ceramic powder would naturally lead to a lesser extent of quartz detected in the XRD analysis, even if the pozzolanic reactivity of both powders was comparable.

#### TGA results

**Fig. 11:**
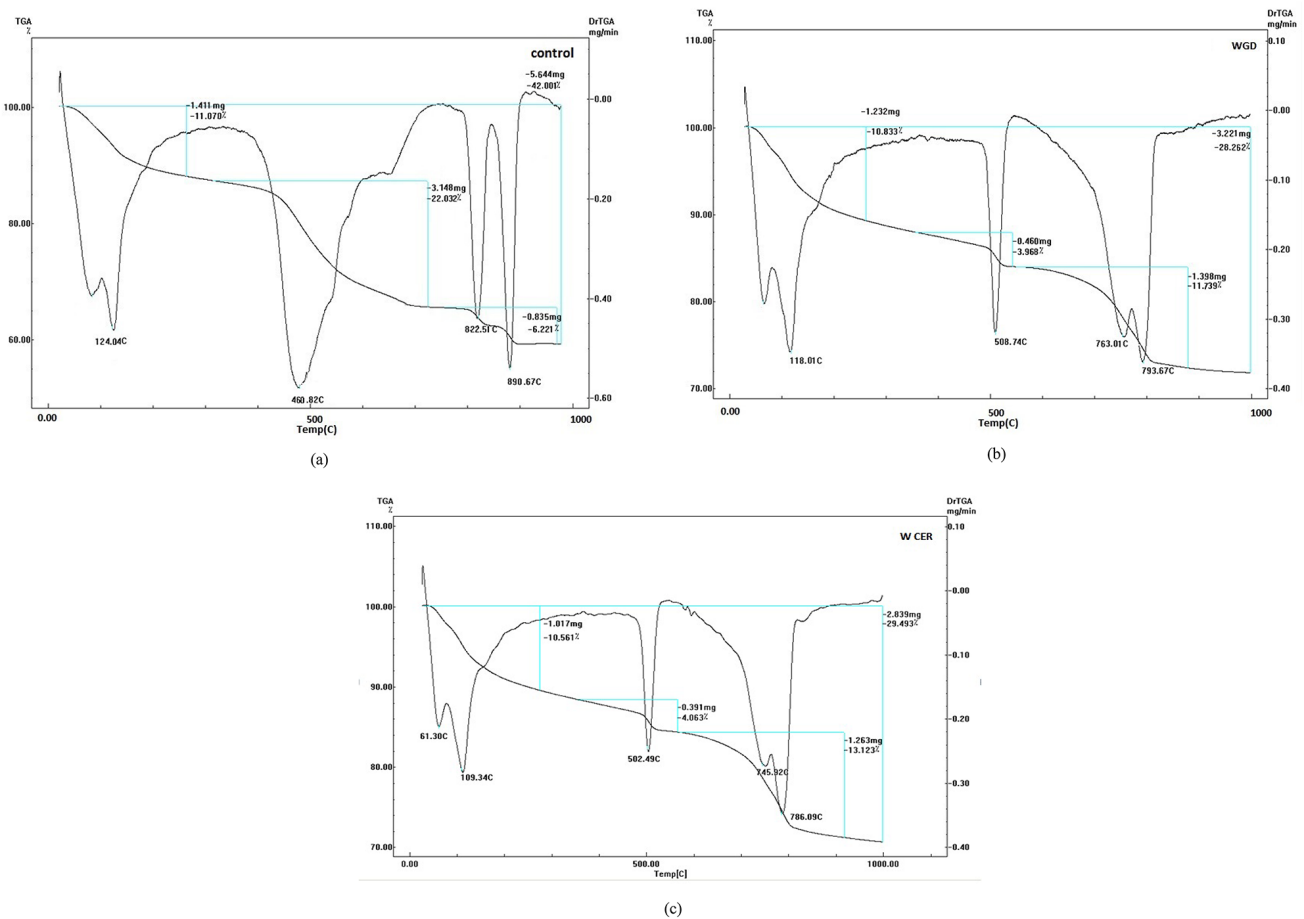
TGA-DTA analysis of (**a**) Control mix, (**b**) 7% WGDP mix, and (**c**) 7% WCP mix.

Fig. 11 illustrates the TGA curves alongside their corresponding differential thermal analysis (DTA) curves for the hydrated samples evaluated at 28 days. The TGA curves display three distinct endothermic peaks, each indicative of the decomposition of specific hydration products within the samples. The initial endothermic peak, observed between approximately 115 °C and 125 °C, is associated with the removal of physically adsorbed water molecules and the dehydration of calcium silicate hydrates (CSH) phases. The second peak, occurring between 460 °C and 508 °C, corresponds to the dehydroxylation of portlandite (Ca(OH)_2_), a typical hydration product of Portland cement. The final peak, detected between 776 °C and 890 °C, represents the decomposition of calcium carbonate (CaCO_3_). Notably, the TGA curves for both the granodiorite and ceramic mixes (Fig. 11b and c) show a higher degree of CSH decomposition in comparison to the control mix (Fig. 11a). This phenomenon can be attributed to the presence of pozzolanic materials in the granodiorite and ceramic mixes. These pozzolanic materials react with the calcium hydroxide released during cement hydration to form additional CSH phases. Consequently, the control mix, which contains only Portland cement and lacks pozzolanic materials, exhibits a lower degree of CSH decomposition due to its higher initial content of unreacted calcium hydroxide. Additionally, the control mix displays the most significant dehydroxylation peak for portlandite, indicating a greater abundance of this phase compared to the other mixes. This can be explained by the relatively higher content of Portland cement in the control mix, leading to a larger amount of calcium hydroxide formation during hydration. Interestingly, all other mixes exhibit comparable decomposition percentages for portlandite, suggesting similar levels of pozzolanic reaction or a potential limitation in the available calcium hydroxide for further CSH formation. The final endothermic peak, indicative of the decomposition of calcium carbonate, is higher for the ceramic mix (Fig. 11c) compared to the granodiorite mix (Fig. 11b). This discrepancy can be attributed to the inherent presence of calcite minerals within the initial composition of the WCP.

#### Density and EDX results

Table 4 provides the values of the density and the elemental composition, measured via EDX analysis, of the optimal concrete mixes (GD7 and C7) containing waste powders at their optimal replacement ratios (7% for both granodiorite and ceramic), compared to the control mix. The measurements were performed for the concrete samples at ambient and elevated temperatures (400 °C, 600 °C, and 800 °C). The incorporation of waste material powders at their optimal ratios generally resulted in a noticeable increase in the density of the concrete mixes. This enhancement can be attributed to two primary factors: denser packing and pozzolanic activity. The finer particles of the waste material powders achieve a denser packing within the concrete matrix compared to the control mix. This improved packing reduces porosity and contributes to a more compact microstructure. Additionally, the waste powders exhibit pozzolanic activity. They react with calcium hydroxide, a byproduct of cement hydration, to form additional cementitious materials. This further contributes to a denser and more compact microstructure. However, exposure to elevated temperatures resulted in a decrease in density for all concrete mixes. This phenomenon is well-documented, as high temperatures induce thermal expansion within the concrete. This expansion can release trapped air and moisture, leading to a less compact structure. Furthermore, the interplay of thermal variations with changes in moisture and air content can also influence the elemental composition of the mixes at different temperatures. The data presented in Table 4 will be utilized to construct a simulation model for evaluating the concrete’s radiation attenuation capability using MCS and PhyX.

**Table 4:** EDX results for the optimum concrete mixes.

Mix	Symbol	Density ρ(kg/m^3^)	O K	Ca K	Si K	Al K	Fe K	Mg K	K K	Na K	C K	Ti K	Temp.
Control	CO	2.44	37.74	42.38	6.63	1.41	1.46	0.22	1.98	1.88	5.99	0.14	Room Temp.
7% WGDP	GD7	2.64	43.39	28.47	7.66	1.94	1.81	0.6	1.99	0.69	13.18	0.17	
7% WCP	C7	2.69	42.38	28	7.59	1.84	3.71	0.56	2.13	0.87	11.74	1.11	
Control	400CO	2.32	35.99	43.58	6.81	1.45	1.72	0.32	2.01	1.94	5.93	0.15	400 ^0^C
7% WGDP	400GD7	2.52	42.12	28.98	7.98	2.03	1.94	0.65	2.04	0.78	13.13	0.22	
7% WCP	400C7	2.59	41.13	28.41	7.79	1.98	3.84	0.62	2.15	0.91	11.89	1.16	
Control	600CO	2.22	34.87	44.08	7.10	1.52	1.77	0.39	2.00	2.07	5.85	0.18	600 ^0^C
7% WGDP	600GD7	2.43	41.18	29.40	8.33	2.14	1.99	0.66	2.01	0.82	13.11	0.23	
7% WCP	600C7	2.48	40.22	29.16	8.1	2.06	3.92	0.55	2.11	0.92	11.62	1.2	
Control	800CO	2.09	33.69	43.75	8.62	1.46	1.80	0.38	1.95	2.03	5.95	0.21	800 ^0^C
7% WGDP	800GD7	2.29	40.54	29.58	8.60	2.24	2.07	0.72	1.95	0.89	13.03	0.24	
7% WCP	800C7	2.37	39.21	29.86	8.29	2.13	4.07	0.62	2.06	0.96	11.42	1.23	

### Radiation shielding measurements

The CM_LAC_ were calculated using the MCS and the PhyX web software at 0.015 ≤ γE ≤ 15 MeV. The values computed by PhyX and the simulated CM_LAC_ values were reasonably close, with a Δ = 3.969%. As the γE increases (at in MeVs 0.015 ≤ γE ≤ 15), the CM_LAC_ of the manufactured CM samples falls. The simulated CM_LAC_ values for the CO group samples drop from 37.425 to 0.057 cm^−1^ for CO, from 36.963 to 0.055 cm^−1^ for 400CO, from 35.919 to 0.053 cm^−1^ for 600CO, and from 33.909 to 0.050 cm^−1^ for 800CO samples. The simulated CM_LAC_ values for the C7 group samples drop from 34.745 to 0.060 cm^−1^ for C7, from 34.479 to 0.058 cm^−1^ for 400C7, from 33.763 to 0.056 cm^−1^ for 600C7, and from 33.039 to 0.054 cm^−1^ for 800C7 sample (Table 5). The simulated CM_LAC_ values for the GD7 group samples drop from 30.915 to 0.058 cm^−1^ for GD7, from 30.422 to 0.056 cm^−1^ for 400GD7, from 29.825 to 0.054 cm^−1^ for 600GD7, and from 28.458 to 0.051 cm^−1^ for 800GD7 sample.

**Table 5:** The linear attenuation values for the CM sample under varying temperatures at different photon energies.

Photon energy (MeV)	CM_LAC_, cm^−1^											
	CO	C7	GD7	400CO	400C7	400GD7	600CO	600C7	600GD7	800CO	800C7	800GD7
0.015	37.425	34.745	30.915	36.963	34.479	30.422	35.919	33.763	29.825	33.909	33.039	28.458
0.030	5.256	4.959	4.424	5.133	4.874	4.312	4.990	4.771	4.224	4.709	4.663	4.030
0.050	1.460	1.429	1.304	1.413	1.393	1.260	1.370	1.357	1.230	1.292	1.320	1.170
0.080	0.627	0.644	0.609	0.610	0.635	0.595	0.589	0.614	0.577	0.555	0.593	0.547
0.100	0.485	0.508	0.488	0.471	0.502	0.476	0.453	0.484	0.461	0.427	0.465	0.436
0.200	0.311	0.338	0.331	0.297	0.328	0.318	0.285	0.315	0.307	0.269	0.301	0.290
0.300	0.262	0.287	0.282	0.250	0.277	0.270	0.240	0.266	0.260	0.226	0.254	0.245
0.400	0.234	0.256	0.252	0.222	0.247	0.240	0.213	0.236	0.232	0.200	0.226	0.218
0.500	0.213	0.234	0.230	0.202	0.224	0.219	0.193	0.215	0.211	0.182	0.206	0.199
0.600	0.196	0.215	0.212	0.186	0.207	0.202	0.178	0.199	0.195	0.168	0.190	0.183
0.800	0.172	0.189	0.186	0.163	0.182	0.177	0.157	0.174	0.171	0.147	0.167	0.161
1.000	0.155	0.170	0.168	0.147	0.163	0.159	0.140	0.156	0.153	0.132	0.149	0.145
2.000	0.109	0.120	0.118	0.103	0.115	0.112	0.099	0.110	0.108	0.093	0.105	0.102
3.000	0.089	0.097	0.096	0.085	0.094	0.091	0.081	0.090	0.088	0.077	0.086	0.083
4.000	0.078	0.085	0.084	0.075	0.083	0.080	0.072	0.079	0.077	0.068	0.076	0.073
5.000	0.072	0.078	0.076	0.069	0.075	0.073	0.066	0.072	0.071	0.062	0.069	0.067
6.000	0.067	0.073	0.071	0.065	0.070	0.068	0.062	0.068	0.066	0.058	0.065	0.062
8.000	0.062	0.067	0.065	0.060	0.065	0.062	0.057	0.062	0.060	0.054	0.059	0.057
10.000	0.060	0.063	0.062	0.057	0.061	0.059	0.055	0.059	0.057	0.052	0.057	0.054
15.000	0.057	0.060	0.058	0.055	0.058	0.056	0.053	0.056	0.054	0.050	0.054	0.051

**Fig. 12:**
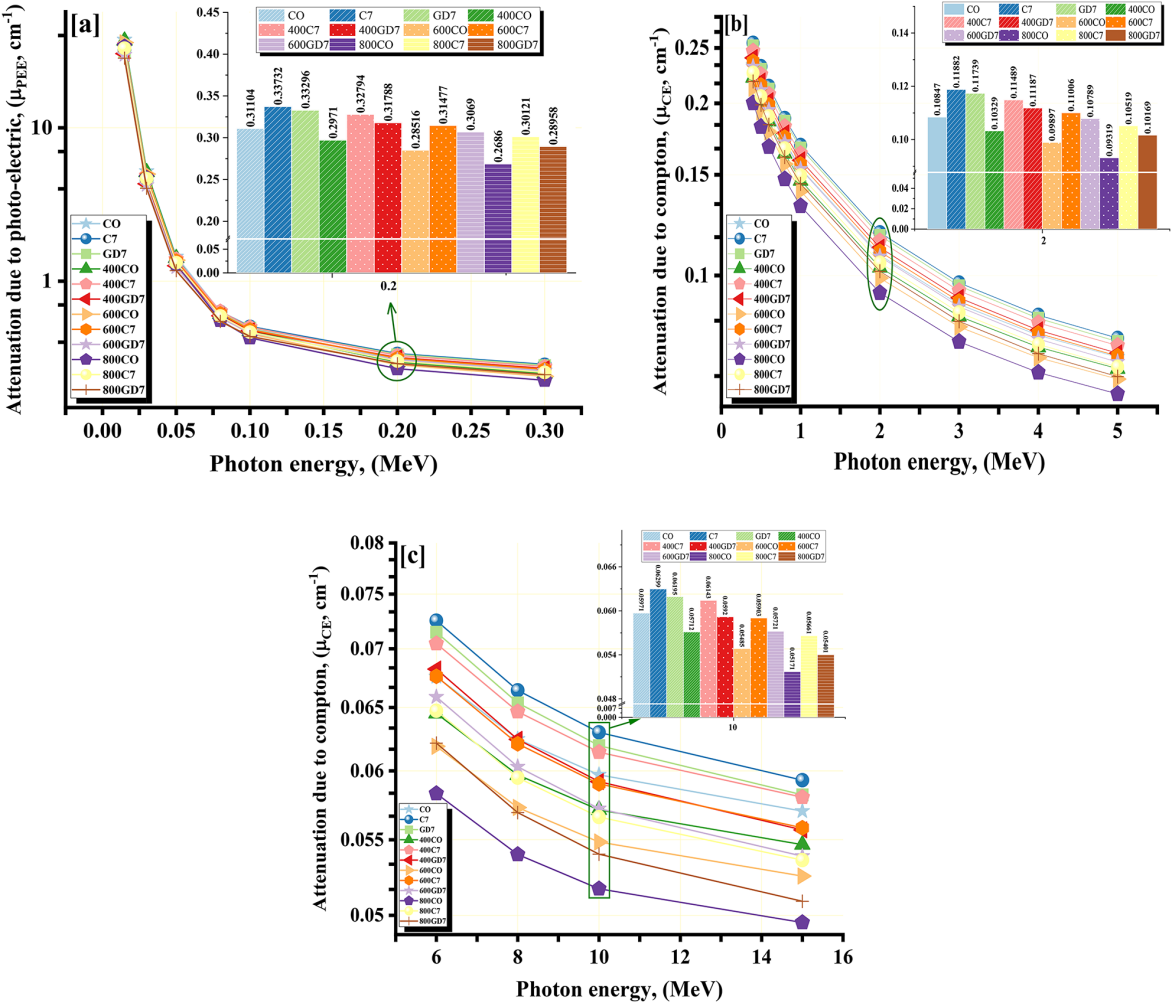
Influence of γ-ray energy on CM_LAC_ (**a**) photo electric, (**b**) and (**c**) compton scattering for the CM-concrete samples.

The photoelectric (PEE) interaction, displayed in Fig. 12a as a drastic drop in the simulated CM_LAC_ values for CM samples, has altered the cross-section (δ) with γE^−4:−5^^52–54^. So, as the enhancement of γE values is correlated with a comparable decrease in the PEE, the interaction δ undergoes a considerable decline. The increase of the applied γE at 0.015 ≤ γE ≤ 0.300 MeV causes an authoritarian exponential decreasing tendency. Fig. 12b and c demonstrate that the virtual CM_LAC_ values experience a rapid decrease in an exponential manner at 0.400 ≤ γE ≤ 15 MeV. The exponential decline is attributed to the changes in δ caused by the compton scattering (CE) interaction with γE^−1^. The lower propensity of higher γ-energy to interact with the atoms of the CM samples is due to their incredible velocity^55^. Consequently, as the levels of energy increase, the probability of γ-absorption decreases while the probability of scattering associated with the γ increases^56^. It was observed that the increase in γE values was linked to a gradual decrease in the δ, which was a consequence of a decrease in the number of γ-electron contacts. This decrease was monitored by a gradual decrease in the CM_LAC_.

**Fig. 13:**
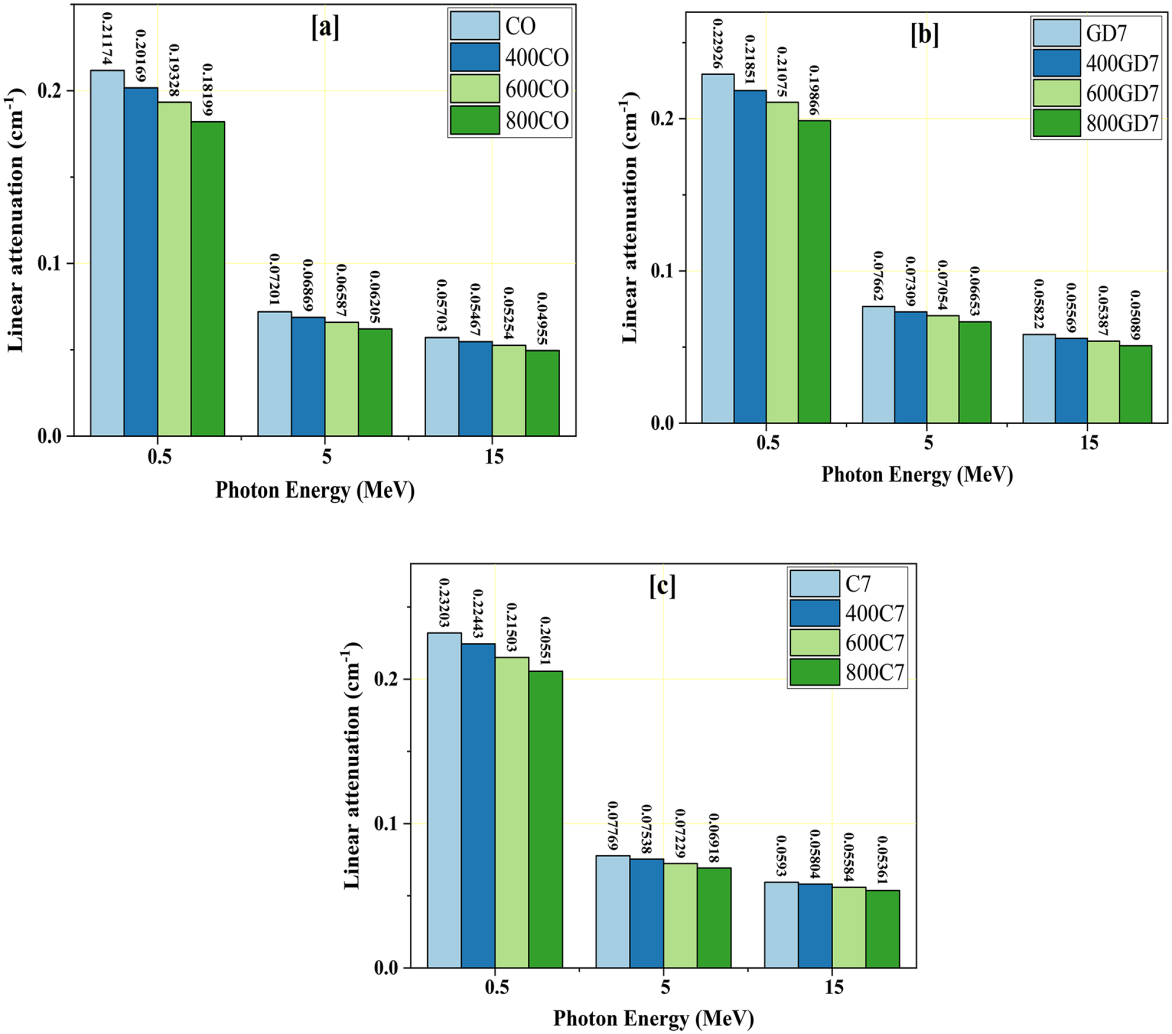
A comparison between the CM_LAC_ at selected γE = 0.5, 5, and 15 MeV and different temperatures.

**Fig. 14:**
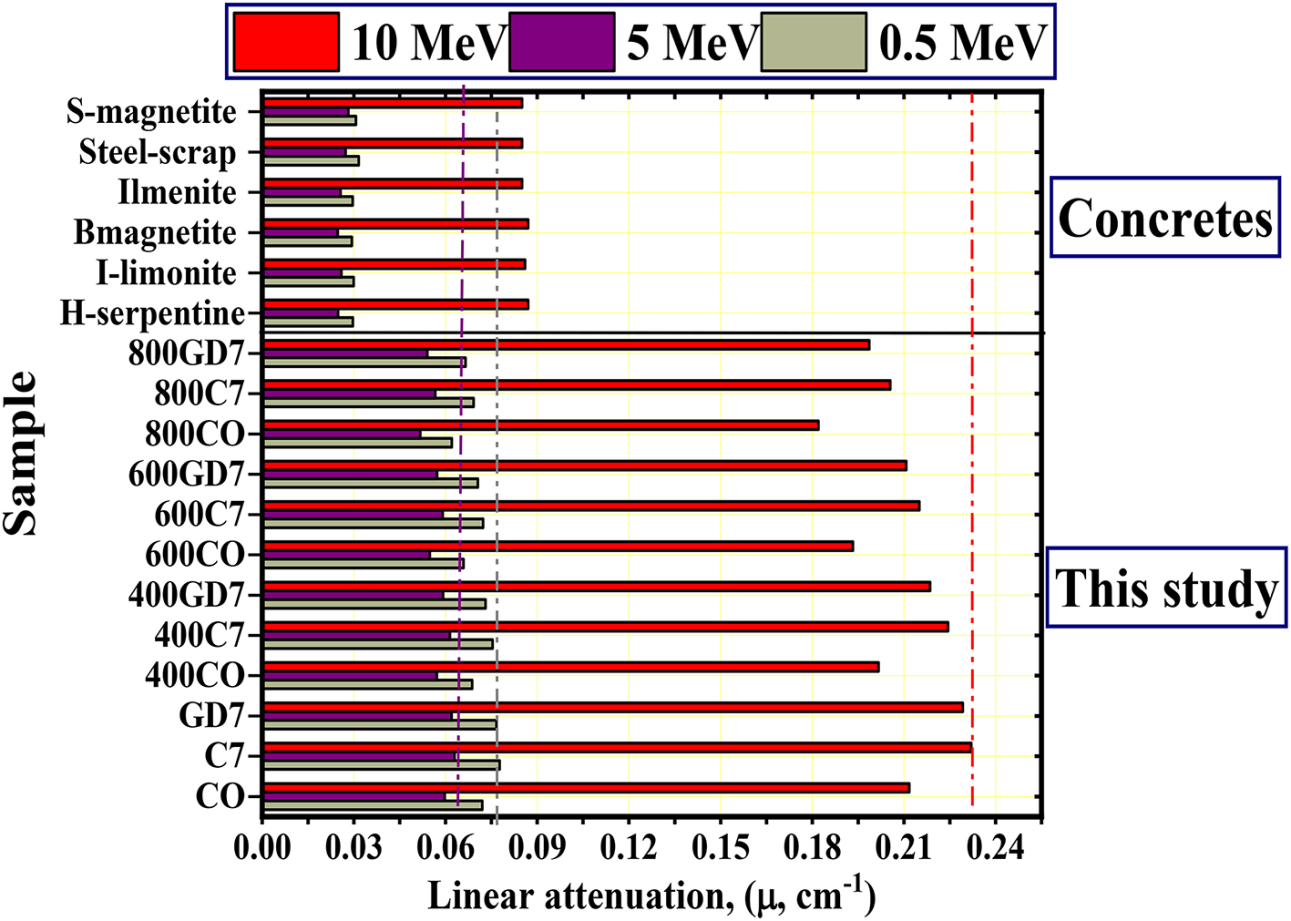
The CM_LAC_ vs. the photon energy for the prepared samples and other concretes.

Also, Fig. 13(a-c) represents the CM_LAC_ at selected energies 0.5, 5, and 15 MeV, which confirms that the CM_LAC_ of the studied samples at room temperature (CO, C7, and GD7) is higher than their counterparts after burning at temperatures 400 °C, 600 °C, and 800 °C. At room temperature, the C7 concrete sample has the highest CM_LAC_, followed by the GD7 sample. This is because the sample has a high density (2.69 kg/m^3^) and contains the highest content of Fe (3.710 wt.%). Also, if the CM samples are compared at the same temperature (at 400 °C, 600 °C, 800 °C), the sample will be 400C7, with the highest CM_LAC_. Also, Fig. 14 compares the CM samples with some commercial concretes ^57^. The investigated CM samples were found to be higher than those compared.

**Fig. 15:**
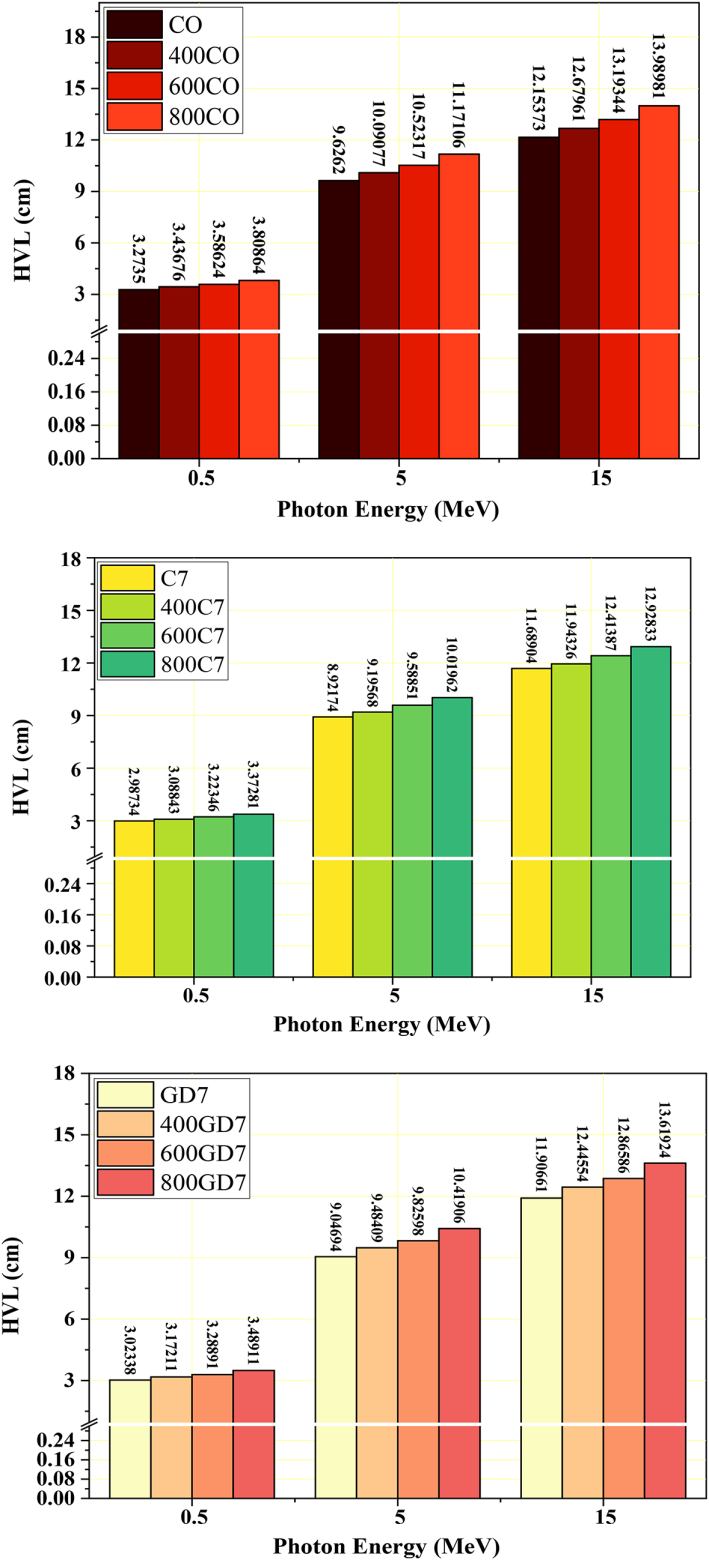
A comparison between the CM_HVL_ at selected γE = 0.5, 5, and 15 MeV at different temperatures for (**a**) CO (**b**) C7, and (**c**) GD7 samples.

Procedures such as the CM_MFP_, CM_HVL_, and CM_TVL _are considered conventional procedures for determining whether radiation shielding is effective^53,57^. In most cases, a lower value for either of the parameters will result in high effective radiative shielding performance^58^. This is due to the fact that radiation is reduced in intensity as it moves through a more restricted area. As the CM_LAC_ values decreased, a consistent increase in the CM_HVL_/CM_TVL_/CM_MFP_ measurement of the CM concrete samples under investigation occurred. The simulated CM_LAC_ values for the CO group samples drop from 0.018 to 12.154 cm for CO, from 0.019 to 12.680 cm for 400CO, from 0.019 to 13.193 cm for 600CO, and from 0.020 to 13.990 cm for 800CO samples. The simulated CM_LAC_ values for the C7 group samples drop from 0.021 to 11.689 cm for C7, from 0.020 to 11.943 cm for 400C7, from 0.021 to 12.414 cm for 600C7, and from 0.021 to 12.928 cm for 800C7 sample. The simulated CM_LAC_ values for the GD7 group samples drop from 0.022 to 11.907 cm for GD7, from 0.023 to 12.446 cm for 400GD7, from 0.023 to 12.866 cm for 600GD7, and from 0.024 to 13.619 cm for 800GD7 sample. Also, the CM_HVL_ values for the CM samples as seen in Fig. 15(a-c). The CM_TVL_ and CM_MFP_ values exhibit a similar pattern to the CM_HVL_ values. The CM_HVL_ / CM_TVL_ and CM_MFP_ values were found to be modified by the ratio of titanium and magnesium to the concretes. Consequently, it is feasible to conclude that the sample with the C7 value displayed the lowest values for CM_HVL_ / CM_TVL_ and CM_MFP_. This indicates that the optimum WCP mix (C7) enhances the attenuation capability of concrete, possibly due to its high density (2.69 kg/m^3^) and significant Ti and Fe (1.11%, 3.71%) content. The GD7 samples comes next and then the CO sample. Also, the temperature effects on the attenuation performance for the Cm samples. This decline can be attributed to thermal effects, including material weakening, decomposition, phase transitions, and loss of structural integrity, which resulted in reduced density and a decrease in heavy metal content.

**Fig. 16:**
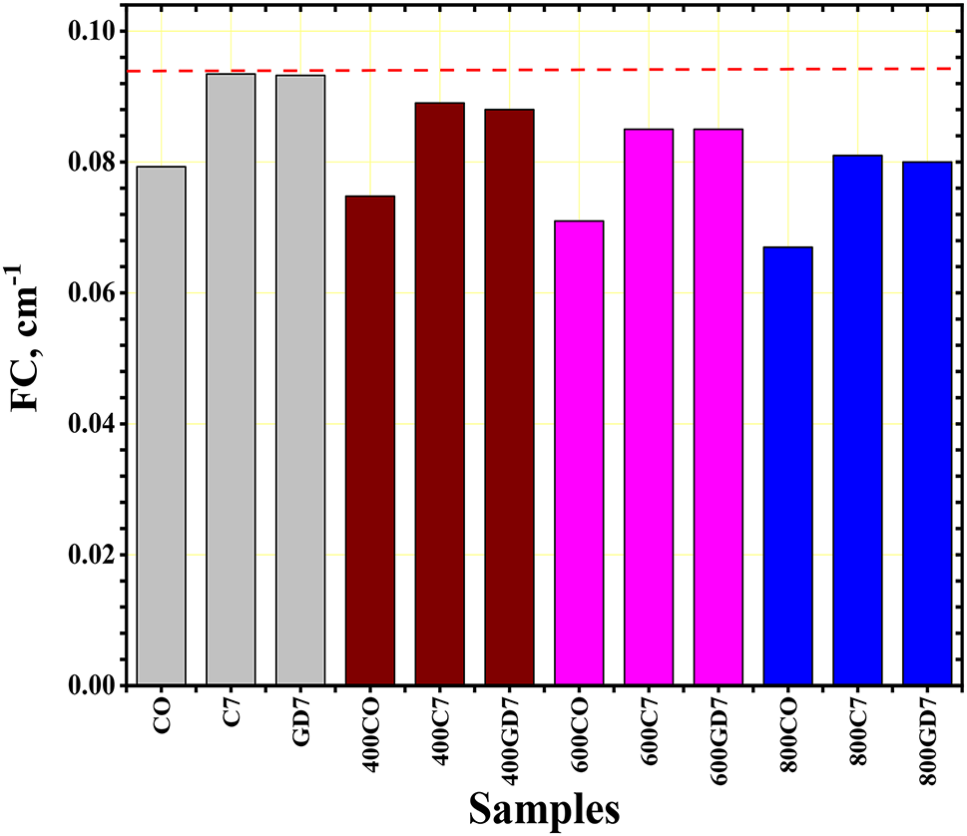
The fast neutron removal cross-section (FC) for the CM samples.

The CM_FC_ of the samples are shown in Fig. 16. The corresponding values for CO, 400CO, 600CO, and 800CO samples were 0.079, 0.075, 0.071, and 0.067 cm^−1^, respectively. The corresponding values for C7, 400C7, 600C7, and 800C7 samples at room temperature were 0.093, 0.089, 0.085, and 0.081 cm^−1^, respectively. The corresponding values of for GD7, 400GD7, 600GD7, 800GD7 samples at room temperature were 0.093, 0.088, 0.085, and 0.080 cm^−1^, respectively (Table 6). The CM_FC_ was present in the C7 group samples because of its high content of oxygen and carbon and its high densities. The CO, C7, and GD7 group samples demonstrated superior FC performance. We can assume that the CM sample under investigation has better neutron shielding characteristics.

**Table 6:** The fast removal cross section for the CM samples.

Sample	Density (Kg/m^3^)	FCS cm^−1^
CO	2.44	0.079
C7	2.69	0.093
GD7	2.64	0.093
400CO	2.32	0.075
400C7	2.59	0.089
400GD7	2.52	0.088
600CO	2.22	0.071
600C7	2.48	0.085
600GD7	2.43	0.085
800CO	2.09	0.067
800C7	2.37	0.081
800GD7	2.29	0.080

The enhanced radiation shielding properties of CM-concrete, particularly the C7 and GD7 mixes, make them suitable for various applications in industries requiring effective radiation protection. In nuclear facilities, these materials can be used for constructing shielding walls, reactor containment structures, and storage units for radioactive materials. In medical facilities, they offer potential for use in radiology rooms, radiation therapy units, and linear accelerator shielding. The combination of high density, higher heavy metal content (e.g., iron and titanium oxides), and improved microstructure contributes to their improved attenuation capabilities against gamma rays and fast neutrons. Additionally, the eco-friendly nature of using waste ceramic and granodiorite powders as partial cement replacements aligns with sustainable construction practices, offering a dual benefit of environmental and functional performance.

Further research and validation under industry-specific conditions, including long-term exposure to radiation and structural durability assessments, will ensure the practical implementation of these materials in such critical applications.

## Conclusions

This study investigates the influence of incorporating two types of finely ground construction waste powders (waste granodiorite powder and waste ceramic powder) as partial cement substitutes at varying replacement ratios on the mechanical and radiation shielding properties of concrete. The investigation encompasses both ambient and elevated temperature exposure conditions. Prior to their inclusion in the concrete mix design, the waste powders were subjected to thorough characterization, including their chemical and mineralogical compositions. The key findings of the study, based on the acquired experimental and analytical data, can be summarized in the following points:

1. All replacement ratios of cement with WGDP and WCP led to improved compressive strength values of concrete at the traditional room temperature. The optimal replacement level for both WGDP and WCP was observed at 7%, yielding improvement ratios of 23.1% and 24.7%, respectively.
2. Exposing concrete specimens containing waste material powders to high temperatures revealed a beneficial influence of the optimal replacement ratios on the residual compressive strength after two hours of heat exposure. At the maximum test temperature of 800 °C, the optimal replacement ratios achieved the most significant improvements. Specifically, a 7% replacement ratio for both WGDP and WCP resulted in 28% and 32.6% enhancements in the residual compressive strength of concrete, respectively.
3. Microstructural analysis of XRD, TGA, and EDX were conducted on the investigated waste powders and the concrete mixes containing the optimal replacement ratios. These analyses provided crucial insights into the chemical and mineralogical compositions of the materials employed. Furthermore, they elucidated the mechanisms responsible for the enhanced mechanical strength observed in the concretes incorporating waste powders. Potential contributing factors include the fine particle size of the powders, their ability to promote a denser and more compact microstructure by filling voids within the matrix, and their pozzolanic activity.
4. In the room temperature, the $\:\text{C}\text{M}$_LAC_ order was C7 > GD7 > CO. This indicates that waste ceramic powder (C7) enhances the attenuation capability of concrete, possibly due to its high density and significant Ti and Fe content, which are favorable for shielding or attenuation purposes. Waste granodiorite powder (GD7) also contributes positively to attenuation, though to a lesser extent than C7. Ordinary concrete (CO) exhibits the lowest $\:\text{C}\text{M}$_LAC_, indicating its baseline performance in linear attenuation.
5. After exposer of varies temperatures, the $\:\text{C}\text{M}$_LAC_ order for the CO group was CO > 400CO > 600CO > 800CO. The $\:\text{C}\text{M}$_LAC_ order for the GD7 group was GD7 > 400GD7 > 600GD7 > 800GD7. The $\:\text{C}\text{M}$_LAC_ order for the C7 group was C7 > 400C7 > 600C7 > 800C7. This decline could be attributed to thermal effects such as material weakening, decomposition, phase transitions, or loss of structural integrity, which led to a decrease in density and the reduction of heavy metal content.
6. The CM_FC_ of the CM-concrete samples have values ranging from 0.079, 0.075, 0.071, and 0.067 cm^−1^ for CO, 400CO, 600CO, 800CO samples, respectively and, 0.093, 0.089, 0.085, and 0.081 cm^−1^ for GD7, 400GD7, 600GD7, 800GD7 samples, respectively, 0.093, 0.088, 0.085, and 0.080 cm^−1^ for C7, 400C7, 600C7, 800C7 samples, respectively. The C7 and GD7 samples exhibit higher CM_FC_ values than the others, likely due to their higher density and significant content of light elements, such as oxygen and carbon.

From what has been concluded, this study paves the way for the utilization of construction waste, especially ceramic and granodiorite waste, in enhancing the properties of concrete towards radiation shielding against gamma rays and neutrons. Furthermore, the exceptional performance of CM mixes compared to ordinary concrete suggests their potential application in radiation shielding for various medical and nuclear facilities.

## Data Availability

All data generated or analyzed during this study are included in this published article.
